# Revisiting the Structure and Chemistry of 3(5)-Substituted Pyrazoles

**DOI:** 10.3390/molecules25010042

**Published:** 2019-12-20

**Authors:** Alina Secrieru, Paul Michael O’Neill, Maria Lurdes Santos Cristiano

**Affiliations:** 1 Center of Marine Sciences, CCMAR, Gambelas Campus, University of Algarve, UAlg, 8005-139 Faro, Portugal; Asecrieru@ualg.pt; 2 Department of Chemistry, University of Liverpool, Liverpool L69 7ZD, UK; pmoneill@liverpool.ac.uk; 3 Department of Chemistry and Pharmacy, Faculty of Sciences and Technology, FCT, Gambelas Campus, University of Algarve, UAlg, 8005-139 Faro, Portugal

**Keywords:** pyrazoles, tautomerism, prototropy, annular tautomerism, reactivity, 3(5)-aminopyrazoles, heterocyclic synthesis, pyrazolo[1,5-a]pyrimidines

## Abstract

Pyrazoles are known as versatile scaffolds in organic synthesis and medicinal chemistry, often used as starting materials for the preparation of more complex heterocyclic systems with relevance in the pharmaceutical field. Pyrazoles are also interesting compounds from a structural viewpoint, mainly because they exhibit tautomerism. This phenomenon may influence their reactivity, with possible impact on the synthetic strategies where pyrazoles take part, as well as on the biological activities of targets bearing a pyrazole moiety, since a change in structure translates into changes in properties. Investigations of the structure of pyrazoles that unravel the tautomeric and conformational preferences are therefore of upmost relevance. 3(5)-Aminopyrazoles are largely explored as precursors in the synthesis of condensed heterocyclic systems, namely pyrazolo[1,5-a]pyrimidines. However, the information available in the literature concerning the structure and chemistry of 3(5)-aminopyrazoles is scarce and disperse. We provide a revision of data on the present subject, based on investigations using theoretical and experimental methods, together with the applications of the compounds in synthesis. It is expected that the combined information will contribute to a deeper understanding of structure/reactivity relationships in this class of heterocycles, with a positive impact in the design of synthetic methods, where they take part.

## 1. Introduction

**Figure 1:**
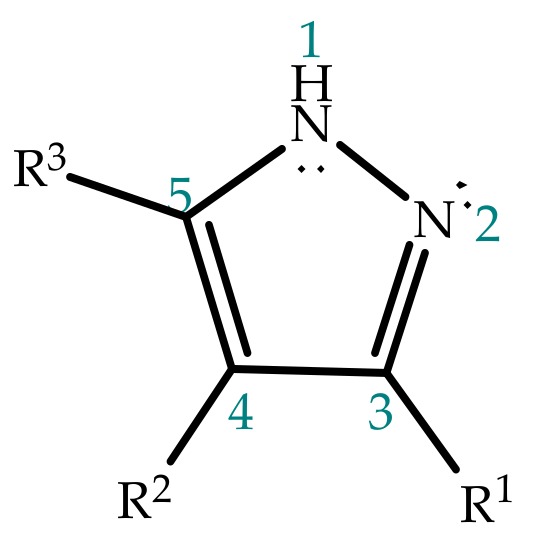
Representation of the general structure of pyrazoles. In the parent pyrazole, R^1^, R^2^ and R^3^ correspond to hydrogen atoms.

Azoles are very important in organic and medicinal chemistry. The multitude of compounds in the azole family provides structural diversity along the various classes of azoles and a wide range of applications in major fields, including in the pharmaceutical industry [1,2,3,4,5,6,7]. Pyrazoles are a class of five-membered heterocycles derived from the parent pyrazole, an aromatic azole with molecular formula C_3_H_4_N_2_ and the structure represented in Figure 1.

The observation that pyrazoles are very rare in nature was ascribed to the presence of a single (-N-N-) bond in their structure, a chemical motif believed to be of very difficult formation by living organisms [8]. Hence, pyrazoles are generally of synthetic origin and serve as building blocks in the synthesis of many other heterocyclic systems, most of which are biologically active, and interesting from a medicinal point of view, rendering this class of compounds worthy of deeper investigations [9,10,11].

The rich reactivity of pyrazoles is linked to their challenging structure, with the possibility of tautomerism [12] and the presence of a multifarious framework, offering versatility for applications in synthetic organic chemistry [13,14]. Derivatives of 3- or 5-amino pyrazole, generally referred to as 3(5)-aminopyrazoles, are especially interesting in this context, serving as starting materials for the synthesis of condensed heterocycles, including pyrazolo[1,5-a]pyrimidines, another privileged motif that attracted interest for many years and is extensively reviewed, in the past and also in recent literature [15,16,17,18]. However, to this date, a thorough investigation regarding the chemistry and diverse reactivity of 3(5)-aminopyrazoles leading to more complex heterocyclic systems has not been performed. A deeper understanding of the structure and chemistry of 3(5)-aminopyrazoles and pyrazoles in general would be beneficial for the elucidation of their chemical potential, their behavior in different environments and how their versatility may affect the development of efficient synthetic methodologies where pyrazoles feature. Several reviews on the chemistry of pyrazoles have been published [19,20,21,22,23,24,25,26,27,28,29,30,31,32,33]; however, these focus mainly on their synthesis and applications, which leaves a void in the reactivity component representative of this class. Therefore, in this manuscript we will focus on the pyrazole moiety, addressing its structure, chemistry and reactivity, with a special attention on its prototropic tautomerism. To showcase the value of pyrazoles as precursors in the synthesis of fused heterocyclic systems, the particular case of 3(5)-aminopyrazoles as building blocks in the synthesis of pyrazolo[1,5-a]pyrimidines will be carefully presented.

## 2. Pyrazole Properties

**Figure 2:**
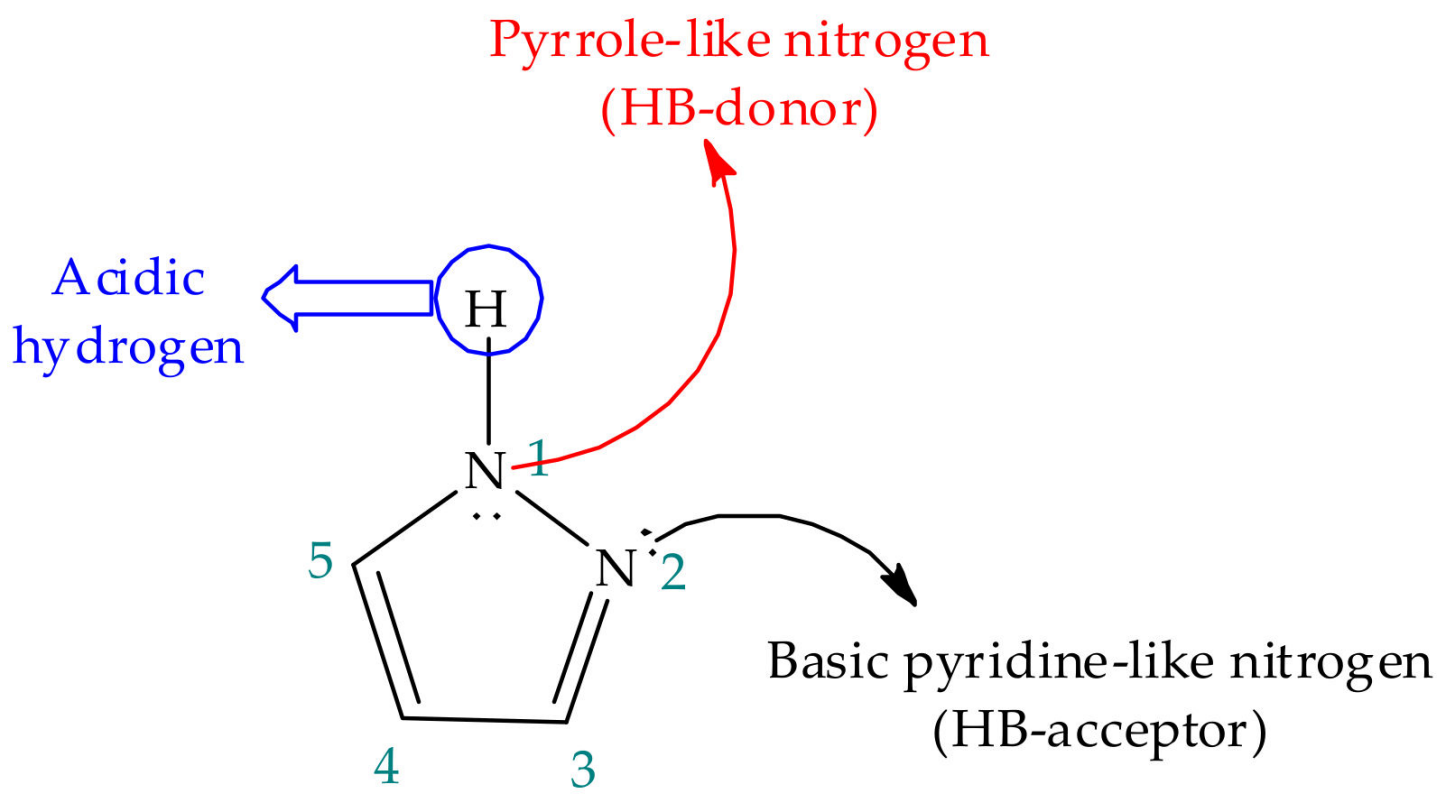
Representation of pyrazole structure, emphasizing the properties of the ring nitrogen atoms.

Pyrazole has a five-membered aromatic ring structure containing two vicinal nitrogen atoms, an acidic pyrrole-like nitrogen with a lone pair of electrons involved in aromaticity, a basic sp^2^-hybridized pyridine-like nitrogen and three carbon atoms (Figure 2) [34], and these combined features must be carefully taken into account in the context of reactivity. In the first place, given the nature of the nitrogen, N-unsubstituted pyrazoles hold amphoteric properties, acting as both acids and bases. While the acidic pyrrole-like NH group easily donates its proton, the basic pyridine-like nitrogen has the ability to accept protons even more readily, and hence, the basic character is generally prevalent. Nevertheless, substitutions on the ring can modulate these properties, as, for instance, electron donating groups were shown to increase the acidity of the pyrrole-like NH group [35,36,37]. Substituent effect and other modulators of proton transfer will be discussed in detail further in this work.

**Figure 3:**
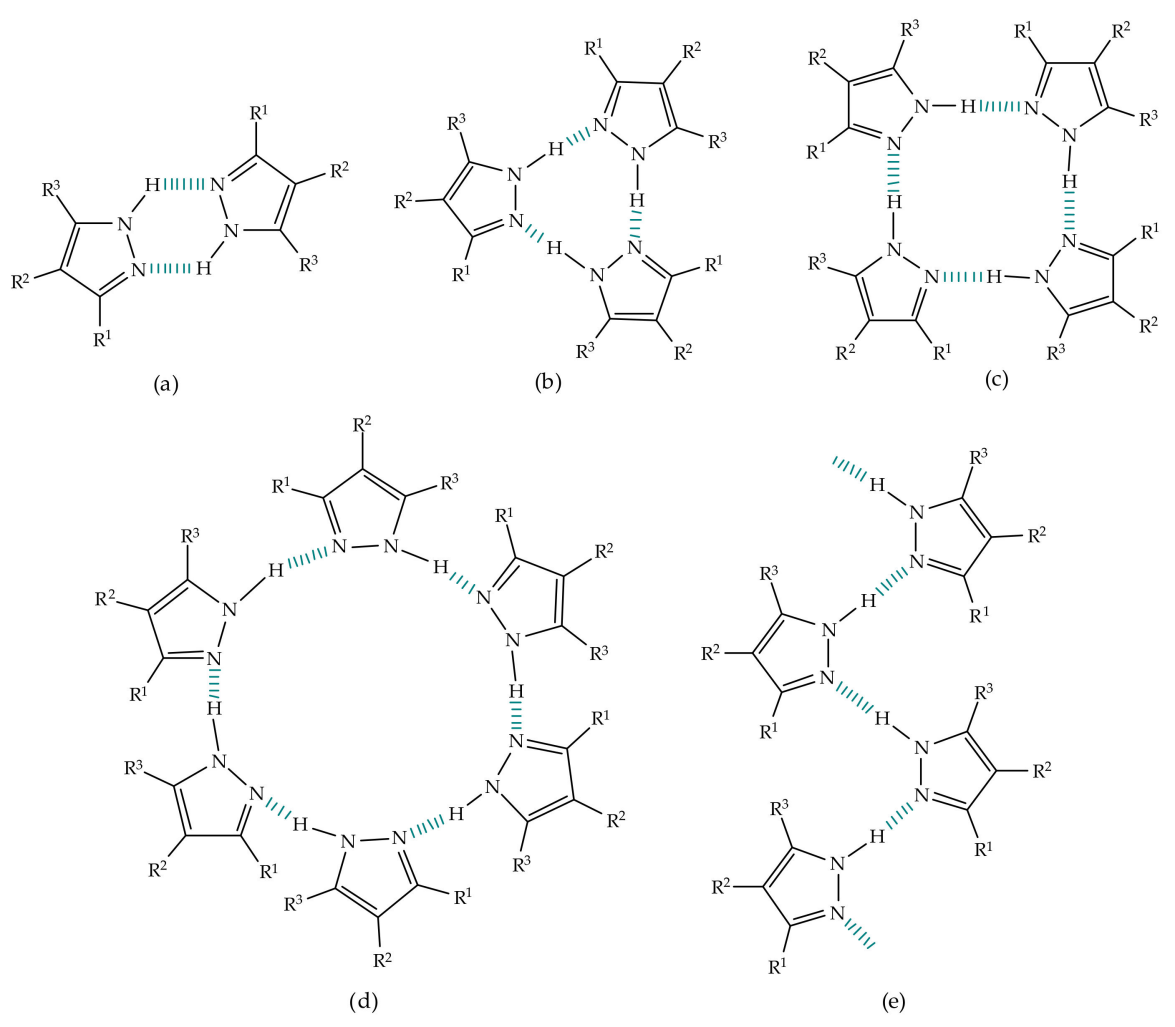
Representation of structures formed from self-association of pyrazole derivatives: (**a**) dimers, (**b**) trimers, (**c**) tetramers, (**d**) hexamers, (**e**) catemers [38,39,40,41].

In addition to the previous, the combination of two dissimilar and adjacent nitrogen atoms in this azole (-N-N(H)- motif) allows it to simultaneously donate and accept hydrogen bonds (HB) (Figure 3), which favors the establishment of intermolecular interactions, either among pyrazole molecules themselves, forming different types of linear and/or cyclic complexes contingent upon the physical state and the nature of the substituents in the ring, or between pyrazoles and neighboring molecules that participate in proton transfer processes [38,39]. Regarding the aggregation pattern of pyrazole in the solid state, X-ray crystal studies unraveled the formation of linear catemers as well as of cyclic dimers, trimers, tetramers and hexamers (Figure 3) [40,41]. In solution, both linear and cyclic oligomers can form, but in this case the associations between pyrazole molecules depend strongly on the type of solvent, since more polar protic solvents can divert the intermolecular interactions towards themselves, favoring the pyrazole-solvent hydrogen bonding rather than formation of pyrazole-pyrazole clusters [38,40]. In the gas-phase, an intermolecular interaction also needs to take place to allow for proton transfer, whether it occurs with another pyrazole molecule or with a third molecule, or even results from collisions with the analytical instrument’s walls [39,42]. Pyrazole-based self-aggregates in the gas-phase have been detected by Infrared (IR) spectroscopy, for the parent pyrazole and for 3,5-dimethylpyrazole, as an equilibrium between monomers, dimers and trimmers [38]. In addition, several theoretical studies were performed regarding intermolecular interactions in pyrazoles, leading to proton transfers in the gas phase [38,43,44].

## 3. Tautomerism in Pyrazoles

Tautomerism is a key feature that stems from the aforementioned ability of pyrazoles to exchange protons. Ludwig Knorr, who discovered the mononuclear pyrazole motif as he stumbled upon a pyrazolone scaffold, during attempts to synthesize quinolones, was also one of the first chemists to contribute to the elucidation of the tautomerism phenomenon, back in the 19th century [1,45,46].

Classically, the concept of tautomerism is associated to a mobile equilibrium between two or more isomeric structures that interconvert easily, with energetic barriers for interconversion below circa 20 kcal/mol, i.e., the different forms may coexist in the same medium [47]. This phenomenon can manifest through several distinct forms, according to the type of exchange present. In heterocyclic systems, two criteria must be considered when discussing tautomerism: the structural aspect of the exchange and the nature of the exchanged element. Regarding the structural aspect, tautomerism is divided into four main types: annular tautomerism, side-chain tautomerism, ring-chain tautomerism and valence tautomerism. In the first type, as the name suggests, the alterations occur within the ring system, between annular carbon and heteroatoms such as nitrogen and oxygen; in the second type, the side-chain elements participate in the interconversion involving the ring; the other two types are characterized by transformations leading to bond formation or rupture—while in ring-chain tautomerism, the migration of the species on the side chain results in ring closure, the valence tautomerism is not a result of migrations of any group or element but rather a rupture or formation of bonds carried by an energetic stimulus [47,48,49,50]. Regarding the type of element involved in the interconversion, tautomerism can be subdivided into prototropy, when the exchange is characterized by the displacement of a proton between two positions suffering alteration, elementotropy, when a heavier element participates in the transformation, frequently a metal atom (metallotropy), and aniontropy and cationtropy, where the two isomers differ only on the position of an anion or cation, respectively [42,49,51,52].

Tautomeric equilibria do not happen solely within one molecule, they can be both intra and intermolecular phenomena. To set straight which kind of process is ongoing, one must take into account the accordance with the thermodynamic and kinetic principles of tautomerism as well as the substitution patterns and the environment conditions to which the compounds are submitted, such as the solvent medium, if present, since solvents usually catalyze isomeric transformations, as the presence and ratio of the tautomers strongly depend on these factors [53]. In other words, many internal and external factors influence tautomeric equilibria, with the dielectric constant of the medium and the temperature also intervening as active modulators of these transformations [47]. It was shown by Alkorta et al. that the proton exchange in pyrazoles is an intermolecular process rather than an intramolecular one, since the energy barrier determined for the intramolecular process displays values surrounding 50 kcal/mol, while the observed values for the intermolecular counterpart do not surpass the range of 10–14 kcal/mol [54]. The information available indicates that protons migrate either with the help of third parties, such as solvent molecules, as for example water molecules were shown to reduce the activation energies when attached to pyrazole, both in the gas-phase and in liquid environment [44], or of other pyrazole molecules, in self-assembled complexes. These contributions will be discussed throughout this manuscript.

### 3.1. Annular Prototropic Tautomerism in Pyrazoles

**Figure 4:**
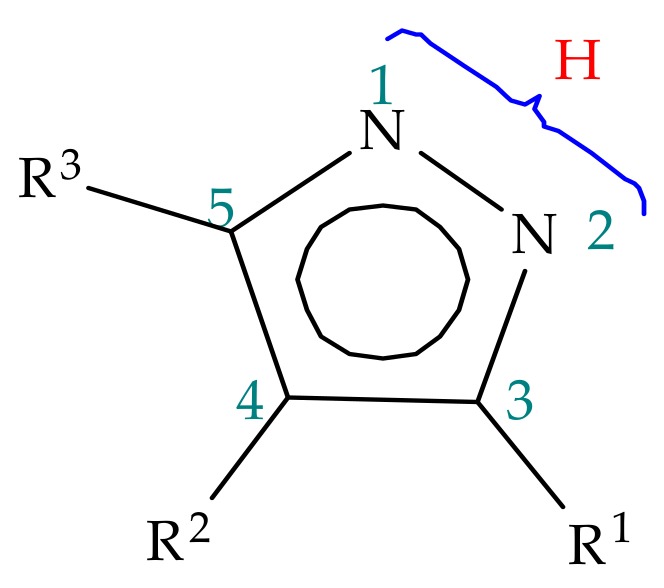
Representation of the two centers involved in prototropy in pyrazoles.

Different types of tautomerism can occur in pyrazoles, depending on the context [12]. In the prototropic annular tautomerism, represented in Figure 4, the NH group in pyrazoles corresponds to the heteroatom within the heterocycle bearing the indicated hydrogen and initiates the numbering of the ring system, with the two nitrogen atoms acquiring the lowest set of locants [55]. As a result, whenever there is a switch in positioning of the hydrogen atom between the two ring nitrogen atoms, a change in structure takes place, which is marked by a shift in numbering at the C3 and C5 carbons, and certainly, changes in properties also accompany these alterations.

Note that when studying tautomerism, one must take into account the physical state in which the analyses are made. Alkorta et al. stated that in the vapor phase, tautomers behave as in a very inert solvent medium, for example a very dilute solution of an apolar aprotic solvent such as hexane [42]. Regarding solid-state and other solvents, similar behavior may be anticipated, but hydrogen interactions must be prudently considered. Moreover, a precept was postulated by Katritzky et al. regarding tautomer stability in the solid state, where higher stability should be expected for the most polar tautomers [56]. In solution, however, tautomeric ratios reflect hydrogen bonding ability and polarizability of the solvating agent, where the dipole moment of the tautomer determines the direction of the equilibrium [36,57].

As such, directed synthesis involving scaffolds that exhibit tautomerism becomes compromised upon the possibility of having two or more different species in the reactant medium, which can be a serious issue for organic and medicinal chemists, affecting chemical industry in general and also drug development. Several theoretical and experimental studies of pyrazole and its derivatives have therefore been undertaken, aiming at the elucidation of the tautomeric structures and their ratios, and will be discussed in the following sections. To facilitate the reader’s interpretation, the studies will be presented in chronological order and each condition will be carefully specified.

#### 3.1.1. Theoretical Studies

With the evolution of science and technology, theoretical studies have earned a vital role in the investigation of physical and chemical properties of atoms and molecules. While in the past chemistry was introduced essentially as an experimental science, nowadays the paradigm is changing and more theoretical studies are needed to complement the experimental knowledge and address current shortcomings [58]. These studies can be performed at different levels of theory, and with such accuracy that, as stated by Elguero et al. [52], in some cases the results obtained can even exceed experimental measurements, also allowing for thorough studies of experimentally unavailable species, such as transition-state species.

Tautomer prediction can be achieved through several types of calculations [53]. Because in pyrazoles there are at least two tautomeric forms arisen from a proton loss or gain, the ratio of each form present in different environments is a function of their equilibrium constants and pKa values. Prediction and determination of the energetic profiles in the studied compounds is an excellent tool for pKa calculation, and therefore, the ratios of each tautomer can be assessed accordingly [58,59]. The idea is to apply the Boltzmann equation for the calculation of isomer ratio in the studied sample, but in this case applicable to an environment in which an acid-base equilibrium can be considered as the phenomenon responsible for interconversion. In that event, Equations (1) and (2) can be applied to attain the needed parameters for tautomeric ratios:

K_T_ = e^−(ΔE/RT)^, (1)

In Equation (1), K_T_ represents the thermodynamic constant of tautomeric equilibrium between hypothetical tautomeric species T_1_ to T_n_, where ΔE is the total energy difference between tautomers expressed in kcal/mol, R is the Boltzmann constant expressed in kcal K^−1^mol^−1^ and T is the temperature expressed in K. Having attained the values for tautomeric equilibrium constants, the percentage of each tautomer is given by the Equation (2) [60]:

%T_1_ = 100K_T_/(1 − K_T_), (2)

Quantum chemistry is often employed in this context, based on Molecular Orbital calculations that lead to parameters such as relative energies, bond distances, bond angles, heats of formation, ionization energies and dipole moments [61]. Ab initio methods, namely Hartree-Fock, configuration interaction (CI) and Møller-Plesset (MP) perturbation theory, are usually the most complex and elaborate type of studies, but one can also apply cheaper and quicker semi- or fully empirical methods for the same purpose [62]. Other types of calculations that may be used involve the density functional theory (DFT) free-energy perturbation, Hammett-type and empirical charge relationships [53]. Density functional theory (DFT) studies, although simpler, emerged as an alternative to ab initio methods, since they are less time consuming and precise enough to be comparable to experimental results, leading in some cases to results even closer to experimental data, and can be applied to many chemotypes, including pyrazoles [63].

##### Tautomer Ratio

Regarding proton transfer in pyrazoles, especially in 3(5)-substituted pyrazoles, recent literature falls short in compiling and exploring in detail the information gathered to date. In early studies, calculations performed on possible tautomers, to evaluate relative stability and abundance, relied on gross approximations, but nowadays the results are progressively becoming more precise. For example, back in 1985, semi-empirical INDO calculations incorporating experimental proton affinity and pKa data allowed Catalán et al. to determine tautomeric ratios of a series of different azoles. In this study, while exploring the basicity of N-methyl or N-unsubstituted 3-, 4- and 5-aminopyrazoles, by means of the tautomeric equilibrium constant in aqueous medium, at 25 °C, they concluded that the 3-tautomer should be more stable than its 5-isomer, with relative abundances of circa 75% and 25%, respectively [64]. Nevertheless, given the available tools at that time, many parameters relied on approximations and interpolations from known compounds, and these results were thus taken into account prudently by the authors.

**Scheme 1:**
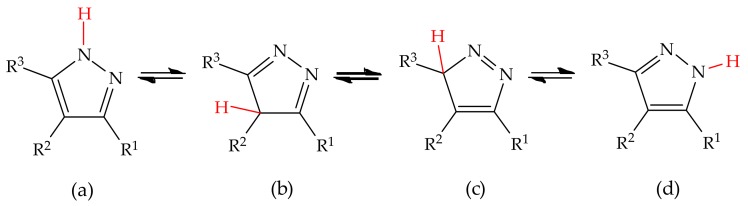
Prototropic rearrrangements on pyrazoles.

In ulterior studies, more precise calculations could be conducted. Technically, there are four possible tautomeric structures for pyrazoles, two involving the nitrogen atoms and the others showing proton occupancy of annular carbons, structures (a) and (d) and structures (b) and (c) of Scheme 1, respectively. Alkorta et al. relied on DFT (B3LYP/6-31G*) and G3//B3LYP calculations to determine whether proton transfer in pyrazoles is restricted to the two nitrogen atoms or if carbon centers are also involved, with a consequent loss in aromaticity. From this study the authors concluded that proton transfer to one of the carbons would require energetic barriers too significant for such migrations to take place, ascribed to loss of aromaticity, confirming the preference for the nitrogen centers in proton exchange [65].

Higher levels of theory (CAM-B3LYP and MP2 methods) applied by Chermahini et al. [35] on the investigation of annular proton transfer in 4-substituted pyrazoles confirmed the data previously obtained by Alkorta et al. [54] with respect to the activation energy for intramolecular proton migration, with calculated values in the range of 47.8–55.5 kcal/mol [35]. The studies by Chermahini et al. have also unraveled the influence of ring-substituents on the overall stability of the system [35]. Results obtained indicated lower energetic demands for proton transfer in pyrazoles carrying electron-donating substituents at the referred position, emphasizing the impact of substitution patterns and electronic effects on tautomer ratio [35].

##### Substituent Effects

Jarończyk et al. performed ab initio MP2/6-311++G** calculations on a range of substituted pyrazoles to evaluate substituent effects on tautomerism [66]. The team varied the electronic nature of the substituents on carbons 3 and 5, the two positions suffering modifications upon proton exchange. The results led the authors to conclude that groups capable of electron donation, mainly through the π-system, such as F, Cl, CONH_2_, NO_2_, OH, NH_2_ and CH_3,_ favor the C3-tautomer, while the electron withdrawing groups BH_2_, CFO, COOH and CHO were found to stabilize the C5-tautomer [66].

**Scheme 2:**
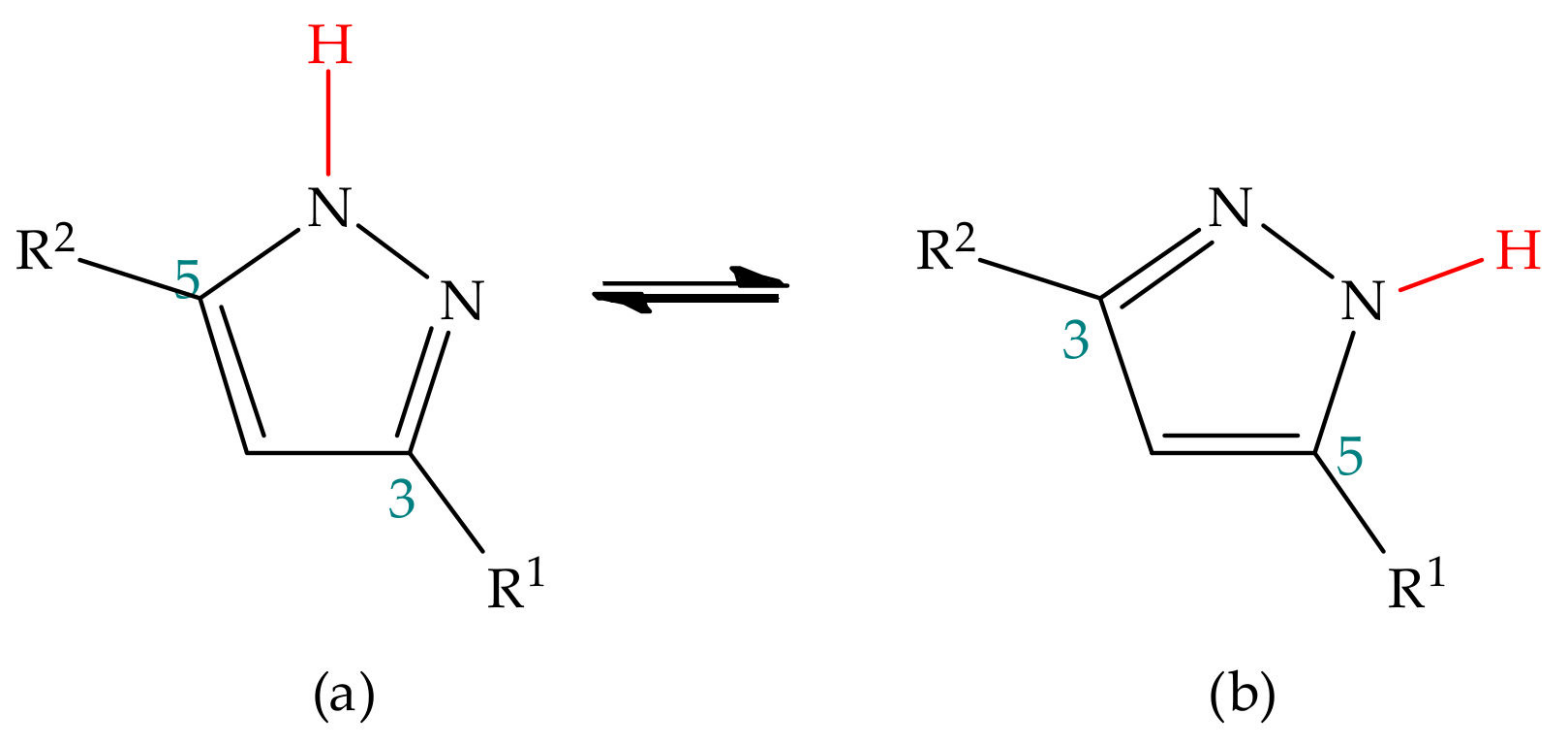
General structures of the pyrazoles 1–6, studied by Claramunt et al. [41].

**Scheme 3:**
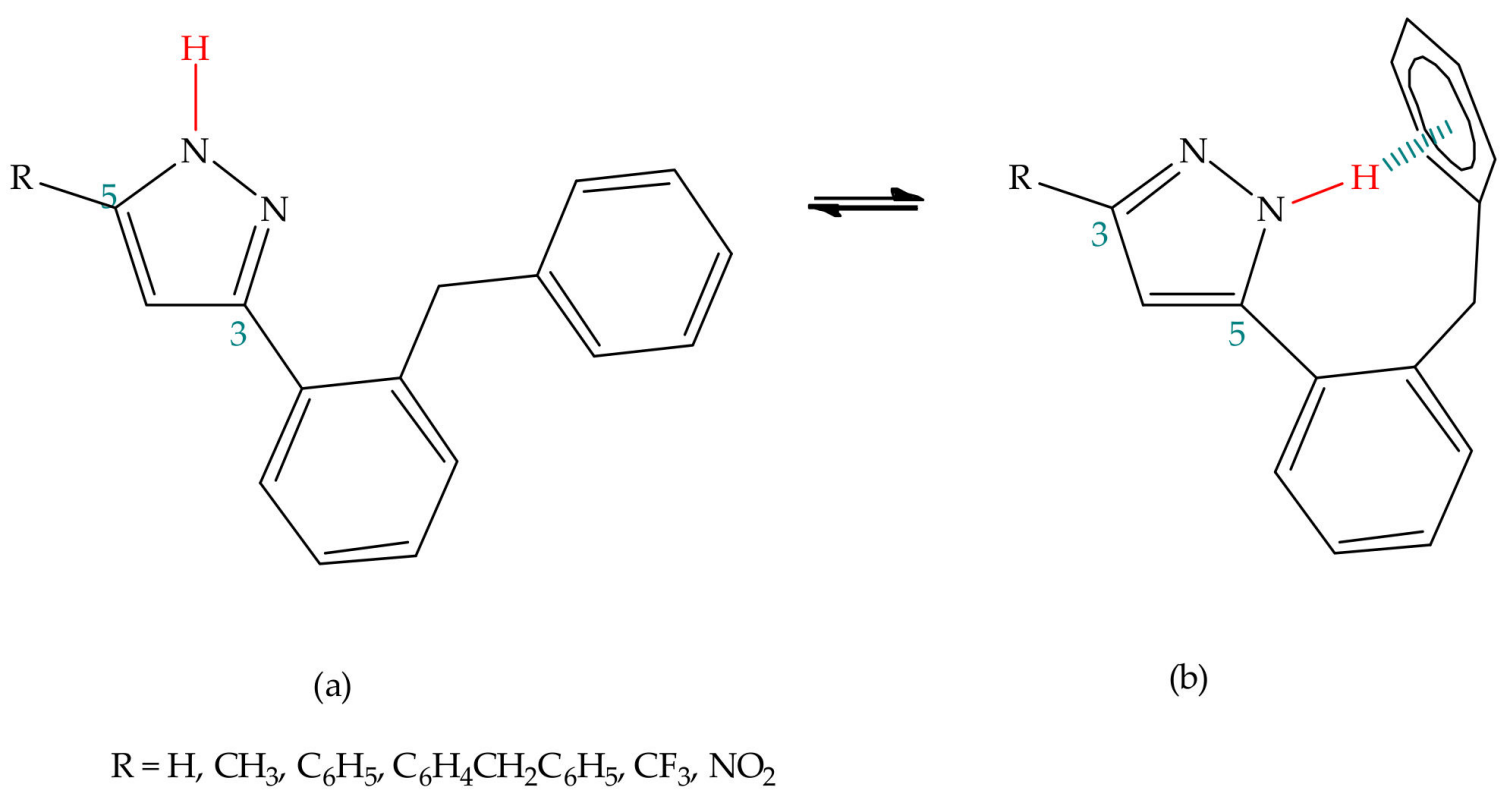
Schematic representation of the tautomeric structures resulting from the C3/C5-benzyphenyl substitution and stabilization of the 5-tautomer through N-H····π interaction, proposed by Cornago et al. [67].

In an attempt to further clarify the effect of substituents on the tautomerism of pyrazoles, Claramunt et al. performed DFT studies at the same level of theory as the above group, B3LYP/6-31G**, on a series of 3,5-disubstituted pyrazoles (Scheme 2) [41]. Results have shown that combinations of phenyl in R^1^ with an alkyl group in R^2^ (methyl, ethyl, isopropyl and benzyl; compounds **1**,**2**,**4**,**5**; Table 1) favor tautomer (a) (Scheme 2), but the introduction of the trifluoromethyl group in R^2^ (compound **3**) switches the preference to tautomer (b), the same applying to the combination of trifluoromethyl and benzylphenyl (compound **6**), which leads to almost exclusive formation of (b) [41]. The authors attributed this behavior to the ability of the benzylphenyl group to establish intramolecular N-H π (phenyl) interactions when attached to C5, causing a torsion and rotation in between the two phenyl rings and a higher stabilization of the overall system (see structure (b), Scheme 3) [41].

The possibility of intramolecular interactions in the presence of the C-benzylphenyl substituents stimulated further investigations on the nature of the tautomeric stabilization. B3LYP/6-31G** DFT and MP2 calculations showed that, besides their ability to modify the torsion angle, directing the phenyl towards the hydrogen on the pyrrole-like nitrogen when attached to C5 (structure (b), Scheme 3), the strength of the bond was highly influenced by the substituents at the 3 position [67]. Upon increase of the electron donating properties of the substituents in R, the tautomeric ratio was shown to switch from almost exclusive formation of (b) to approximately equal amounts of both forms, as shown for R = phenyl. Interestingly, NO_2_ substitution in R determined the highest destabilization of the N-H····π hydrogen bond, as plausibly both nitro and benzylphenyl groups competed for position 5 owing to the possibility of hydrogen bond establishment between the NO_2_ group and the pyrazolic N-H [67].

A similar effect was observed for other pyrazoles bearing a substitution pattern prone to interact intramolecularly. In this case, interactions in study were between C-carboxyl or methoxycarbonyl groups and the hydrogen at N1, which were also shown to stabilize the less stable 5-tautomer [68].

In a continuation study, Alkorta et al. compared the theoretical chemical shifts of three distinct azoles carrying the same substituents [69]. Methyl, phenyl and *t*-butyl groups at C3 and C5 were evaluated to a greater extent with respect to the depth of the applied basis set, using DFT B3LYP/6-311++G(d,p) calculations to determine the structures. Among the pyrazoles studied, aside from the substituent electronic contributions to the nucleus, which favor the occupation of C3 by the electronically dominant group, results also pointed to a higher stability for the azoles carrying the bulkier groups on C3, whether the ring was mono- or di-substituted, at C3 and C5 [69].

These conclusions were later corroborated by Chermahini et al., using the same type of study, through DFT B3LYP and also ab initio MP2 calculations [43]. Dealing with hybrid functionals, at the 6-311++G(d,p) level of theory, the authors reported that 3(5)-substituted pyrazoles detain preferential structures in behalf of the electronic features of substituents: electron donating groups sustain a C3 configuration, while electron withdrawing ones stabilize the system when occupying a C5 position. Furthermore, they could also unravel other interesting properties within the pyrazole scaffold, namely those related to intra and intermolecular proton transfer. Results indicated that intramolecular proton migration in 3-substituted derivatives is favored by the presence of electron donating groups. As for intermolecular interactions, the effect of the substituents was also shown to be quite relevant, since the hydrogen-bond strength in each tautomer was shown to differ according to the electronic character of the attached groups: electron donating groups stabilized 3-substituted dimers, while electron withdrawing ones favored self-associations of 5-substituted tautomers. Additionally, as stated previously in this review, energy barriers determined for intermolecular processes were shown to be lower than the intramolecular ones [43].

Marín-Luna et al. studied the proton affinity and pKa values of a structurally diverse library of pyrazole derivatives and were also able to determine tautomeric ratios in the gas phase and in aqueous solution [70]. Theoretical (DFT) calculations on a library comprising 150 pyrazole derivatives, using the B3LYP functional and the 6-311++G(d,p) basis set, unraveled differences in tautomeric stability upon modifications in ring substituents. A relationship between C3 occupation and the electron donating capacity of the substituent in the studied population of azoles, in the gas phase, could be established. Electron-donating groups showed preference for that position, although slight changes were observed in the presence of substituents at the 4 position, including inversions in ratio in some cases. Strong electron donating groups at C4 seem to promote a tautomeric equilibrium, with results showing an increase in the proportion of the 5-tautomer. In water, although for the majority of compounds under study the tautomeric preferences persisted, a tendency towards equilibrium, or even changes in ratio, was observed [70].

**Scheme 4:**
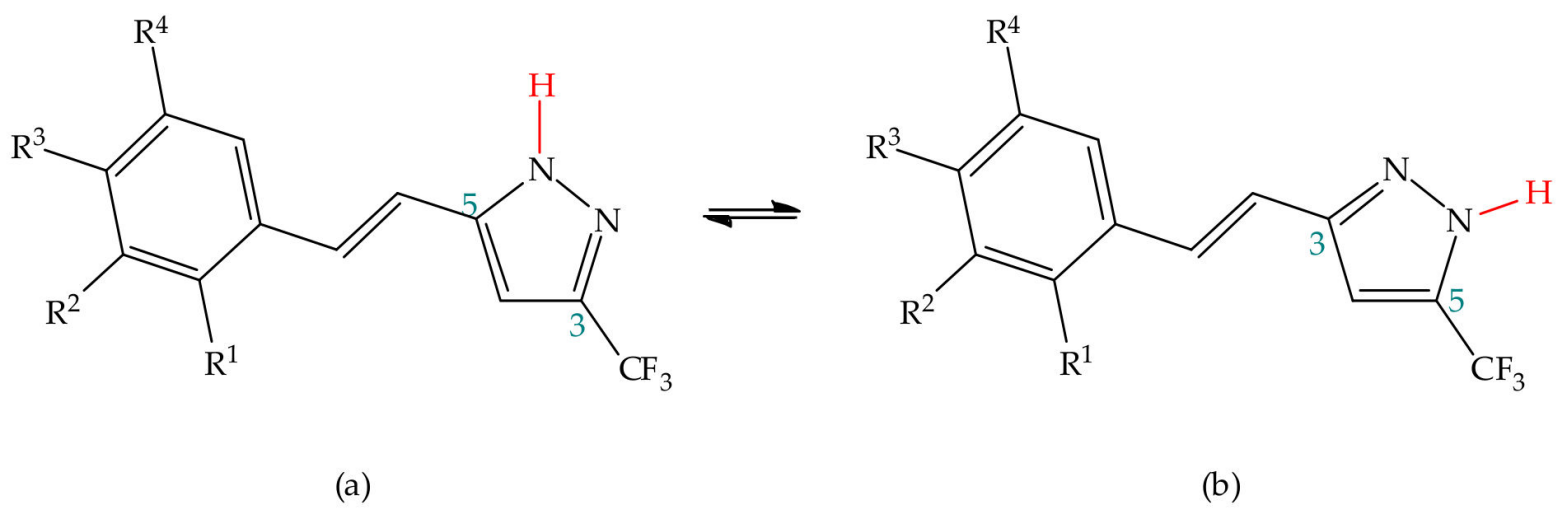
General structures of the 3-CF_3_- (**a**) and 5-CF_3_- (**b**) hemicurcuminoid pyrazoles studied by Nieto et al. [71].

While also addressing this issue, Nieto et al. performed an investigation on pyrazoles derived from hemicurcuminoids incorporating a trifluoromethyl moiety at tautomeric C3/C5 positions (Scheme 4). The study used GIAO/B3LYP/6-311++G(d,p) calculations to assess the effect of the electronic nature (H, F, OH and/or OCH_3_) and substitution pattern on the phenyl group of the hemicurcuminoid styryl substituent on the stability and tautomeric ratios of the different compounds. Results were in agreement with experimental data collected from NMR spectra, showing a higher stability for the tautomer carrying the CF_3_ group at C3, independently of the substituents on the phenyl ring [71].

**Scheme 5:**
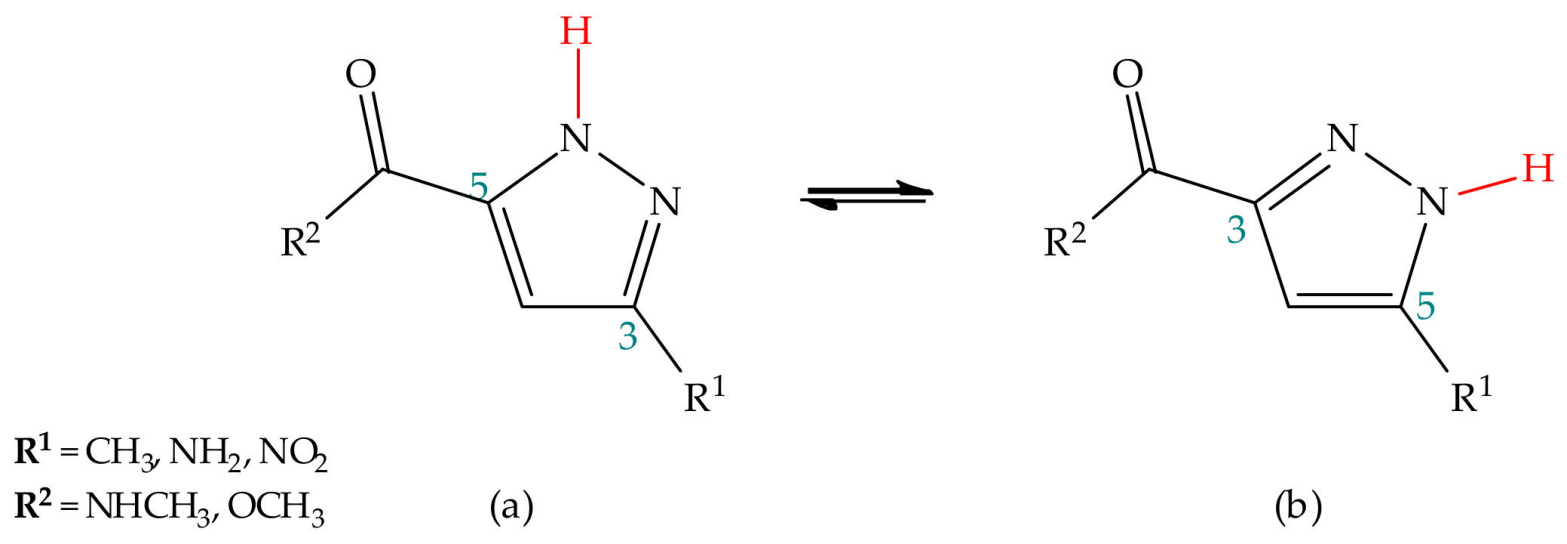
General structures of the ester/amide-linked pyrazole derivatives studied by Kusakiewicz-Dawid et al. [72].

Recently, Kusakiewicz-Dawid et al. explored the annular tautomerism of a set of 3,5-disubstituted pyrazoles incorporating an ester or amide group, aiming at an evaluation of the influence of substituents and the environment on tautomerism and conformation of a small group of 3(5)-amide/ester carbonyl-linkage pyrazole derivatives carrying methyl, amino or nitro groups at the other position (Scheme 5). The conclusions were in much accordance with insofar statements: preferential occupancy of the C3 position is chosen by the most electron donating substituents and the opposite effect is noticed with respect to the electron withdrawing ones; inversion in tautomerism in case of possibility of intramolecular interactions, in most cases between HB acceptor from the carbonyl-linkage derivative and the N1 hydrogen; and also a considerable impact of the environment conditions in tautomeric stabilization is observed for the majority of species, except for those that are stabilized by intramolecular bonds [72]. As such, in addition to substituent effects, the environment conditions interfere on the tautomer ratio in pyrazoles [72], thus deserving close attention.

##### Environmental Effects

When addressing the environment conditions many factors can be included, such as the physical state of the sample, the temperature to which it is submitted and/or the solvent in which it is solvated, whenever applicable. For solutes, we know that tautomer stabilities and equilibria vary according to the solvent’s properties, namely polarity and proticity. A more polar tautomer should be more stable in a polar environment and a more protic solvent is expected to assist to a higher extent the proton transfer reactions [36,57]. Thus, solvation properties of molecules have acquired relevance in the assessment of chemical behavior, not only because many experimental techniques rely on solvation of compounds but also because biochemical processes take place in the condensed phase. In the same way, apart from solvent contribution, all of the intermolecular interactions with the surrounding environment should be considered whenever hydrogen donation and acceptance processes are possible, independently of the physical state. Theoretical studies, although relying on gas-phase behavior, can also be used to predict solid-state and solution properties, due to the possibility of introducing features inherent to different environments in the calculations, and also because pyrazoles preserve their propensity to aggregate in the gas phase, a feature common to all of the physical states [73,74]. Due to this fact, theoretical studies were shown to display high accordance with experimental results in many of the studies reported herein.

**Scheme 6:**
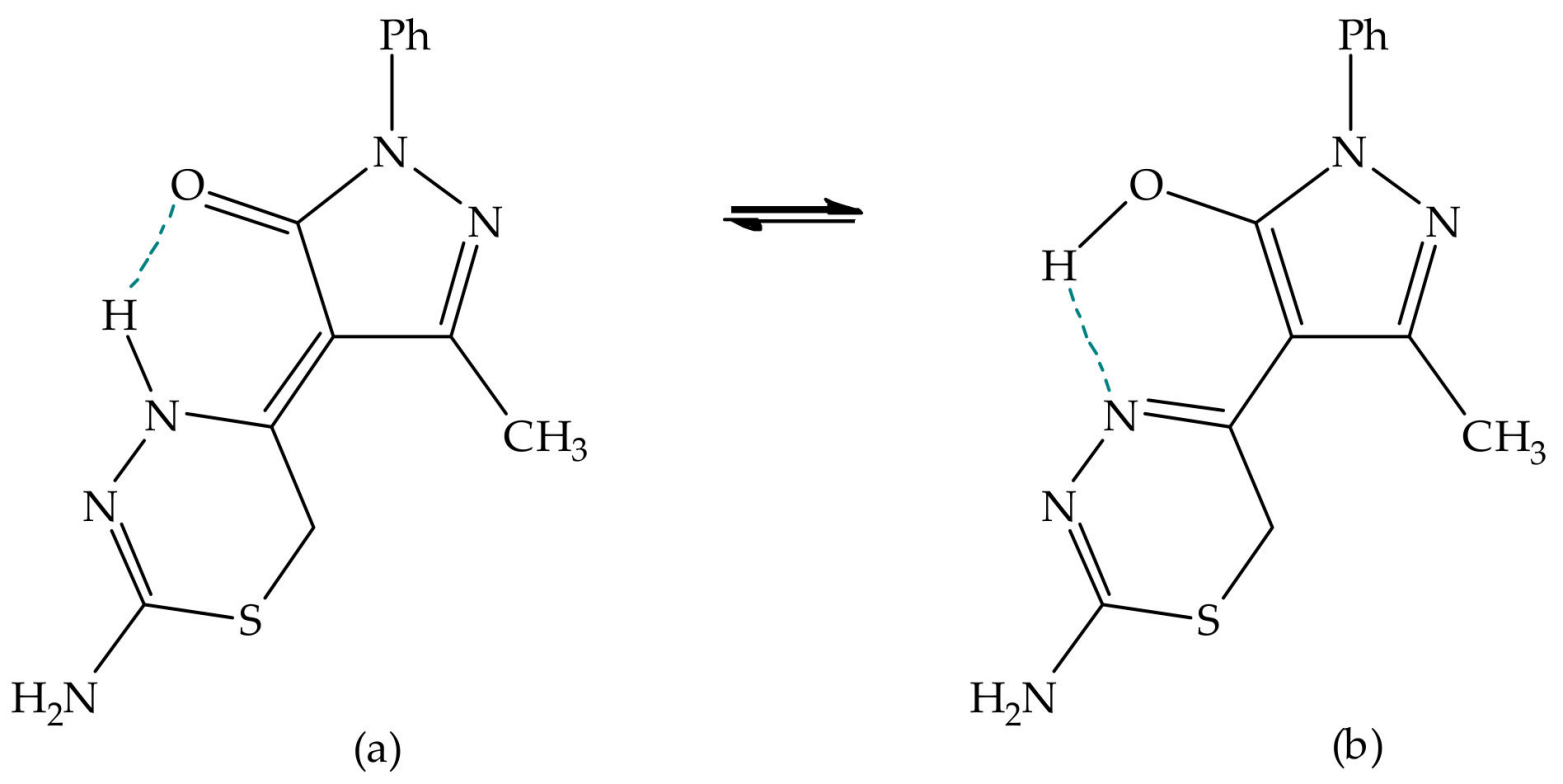
Schematic representation of the keto (**a**) and enol (**b**) tautomeric structures formed from intramolecular hydrogen-bonds in the pyrazole derivatives studied by Lin et al. [63].

A restricted Hartree-Fock and DFT studies were used to investigate the role of solvents in the tautomeric stabilization of 1-phenyl-3-methyl-4-(6-hydro-4-amino-5-sulfo-2,3-pyrazine)-pyrazole-5-ones by Lin et al. [63]. Although in this work the authors did not address the annular tautomerism of pyrazoles, they showed that in pyrazolones which bear the ability to interact intramolecularly through hydrogen bonds, keto-enol tautomerism may arise (Scheme 6). This phenomenon is of exceptional relevance in organic chemistry, owing to its significant prevalence in other heterocyclic systems prone to keto-enol tautomerism by virtue of intermolecular interactions, as shown by our group in studies of quinoline derivatives [75,76,77]. The investigations of Lin et al. revealed that tautomeric stabilization of the pyrazolone system was significantly compromised by the nature of the solvent [63]. Tetrahydrofuran (THF), methanol and ethanol solvents were evaluated, and higher stability was shown for the keto tautomer in all of the studied solvents. Nevertheless, the stability of both tautomers was shown to increase when increasing solvent polarity, whereas the intramolecular hydrogen bonds were shown to become weaker. These alterations were ascribed to molecular dipole moment modulation by the solvent, evidencing the significance of solute polarization by solvent molecules on tautomeric ratios [63].

Nagy et al. determined, at B3LYP and MP2 levels, with the 6-31G* and 6-311++G** basis sets, the effects of four solvents (chloroform, methanol, acetone and water) on the intramolecular proton transfer, in a series of different azoles, reaching similar conclusions to those obtained by Lin et al. [63] regarding the impact of polarity on transformations in solution. Solvent polarity is in intimate relation with the dielectric constant of the molecules, thus, higher solvent polarity results in higher solute polarization. This phenomenon increases the interaction between solute and solvent when a molecule is more polarizable, which proved interesting in tautomer studies since tautomers may be very different structurally and one can be favored over the other according to its polarizability in a specific medium. In this study, pyrazoles showed to be less sensitive to this factor comparatively to the other azoles, since both tautomers were detectable in the wide range of solvents studied [78].

**Figure 5:**
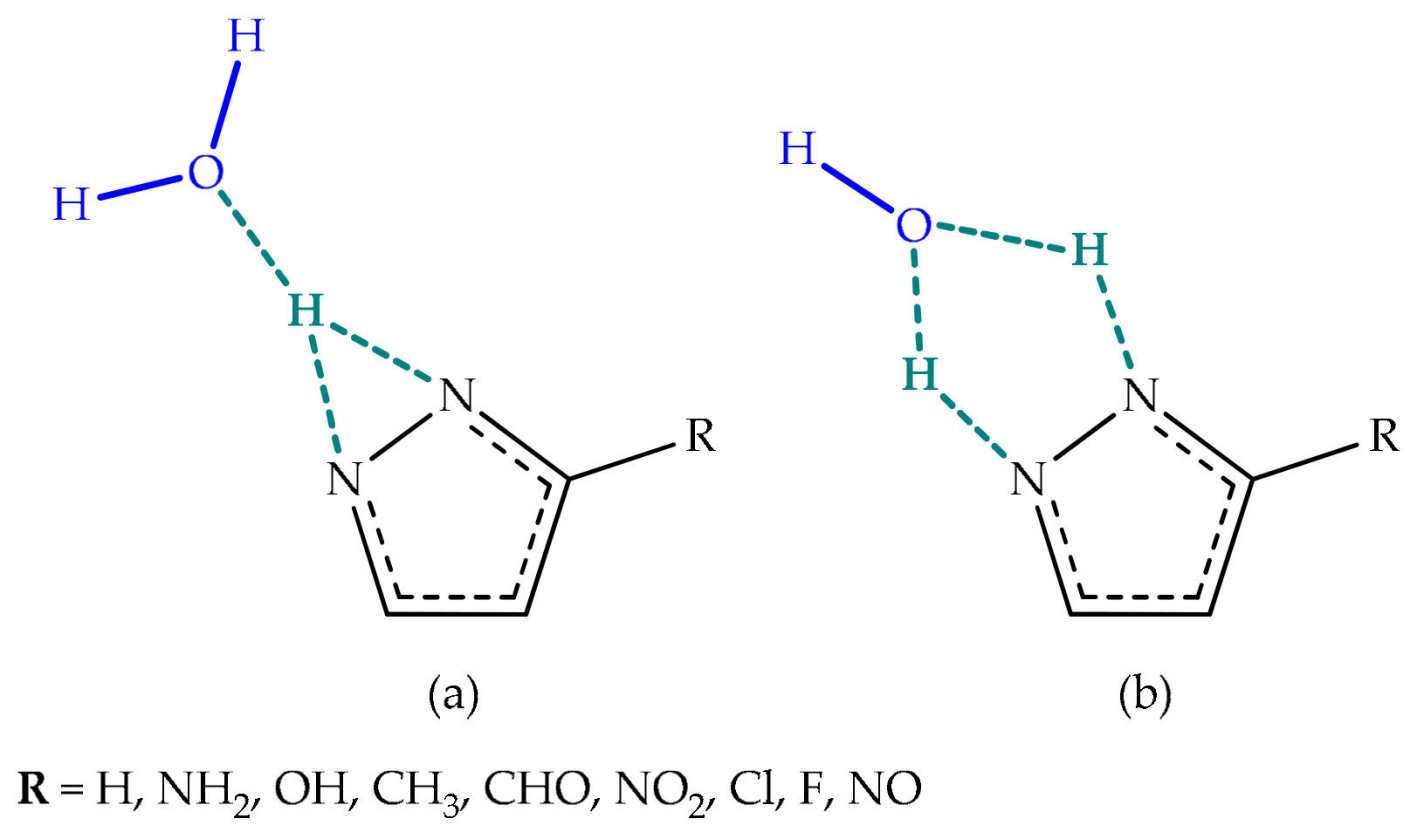
Schematic representation of the transition states resulting from the two types of intermolecular interactions between pyrazoles and a water molecule, evaluated by Chermahini et al. [43].

A computed study led by Beni and Chermahini incorporating a higher basis set (6-311++G(d,p)) showed the significant contribution of solvent molecules in the intermolecular proton transfer and tautomerism of 1*H*-pyrazole-5-thiol [79]. This compound is characterized by the presence of additional tautomeric structures, supplementary to the 3(5)-substituted 1*H*-pyrazoles discussed in the present paper, owing to the possibility of participation of the thiol substituent in side-chain tautomerism. Interestingly, in the gas-phase the products of prototropic annular tautomerism were those leading in terms of stability. As for solution studies, four solvents were evaluated (THF, dimethylsulfoxide (DMSO), ethanol and water) and the results obtained using the DFT and MP2 methods show an overall stabilization of the tautomers with higher dipole moments upon increase of the solvent polarity, in which case it is also worth mentioning that the solvent polarity displayed a key-role in polarization of the solutes [79]. Furthermore, since literature states that hydrogen-bonding entities contribute to the reduction of activation barriers in the tautomerism of pyrazoles, Chermahini et al. was also inspired to scrutinize this feature in a posterior study. Calculations were performed at B3LYP/6-311++G(d,p) and MP2/6-311++G(d,p) levels of theory and two types of solvent molecules were considered, water and ammonia. The main goal was to evaluate the manner in which solvent molecules may interact with pyrazoles leading to proton exchange, and their level of contribution to tautomer activation barriers. Figure 5 depicts the transition states for the two proposed manners of interaction with water molecules, the same being applicable to ammonia. From their study, the authors concluded that lower energetic barriers were achieved for the transition state (b) of Figure 5 and also that ammonia molecules stabilize the transition state to a greater degree than water [43].

**Figure 6:**
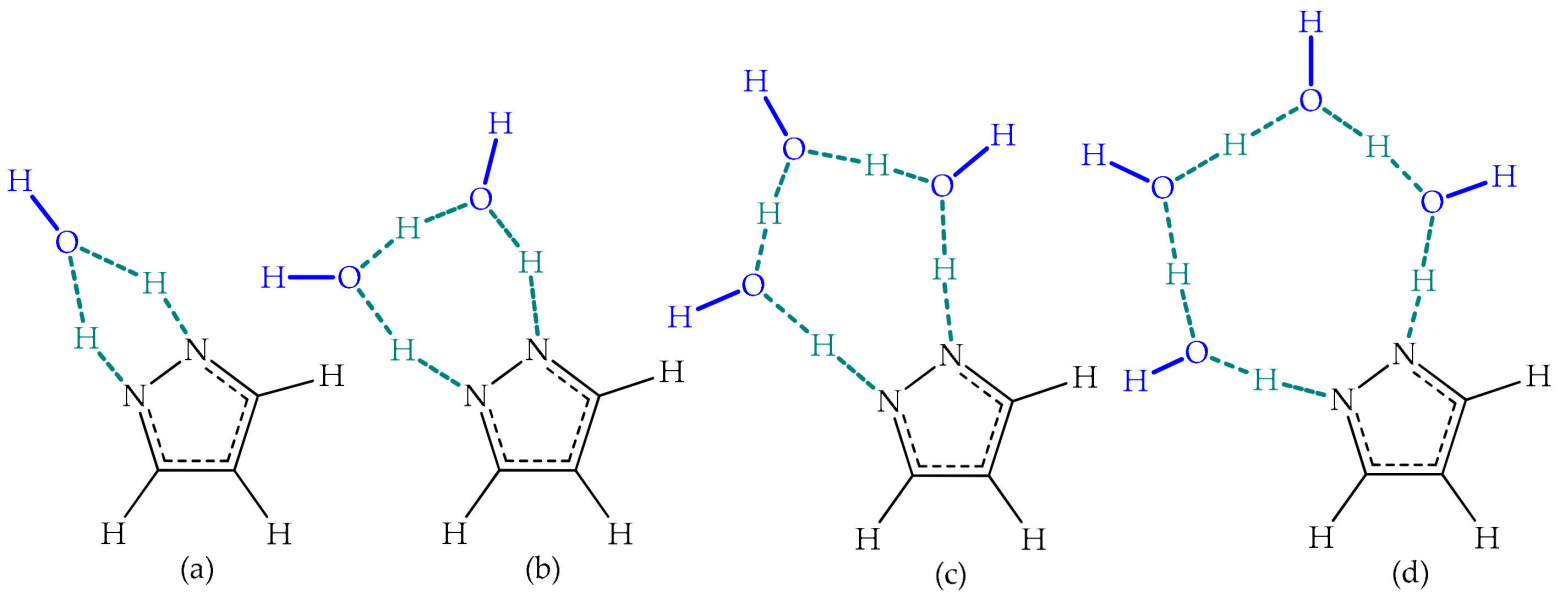
Gas phase MP2 optimized transition states for 1, 2-proton transfer in complexes of pyrazole with (**a**) one; (**b**) two; (**c**) three and (**d**) four water molecules obtained by Oziminski [44].

A study by Oziminski followed, focusing on water-aided proton transfer. The author used ab initio Moller–Plesset MP2/aug-cc-pVDZ and density functional theory B3LYP/6-311++G(d,p) methods and evaluated the kinetics of the 1,2-proton transfer in five different azoles, pyrazoles included, with the aid of 1 to 4 water molecules (Figure 6). The main conclusions regarding pyrazoles were that water lowers energetic barriers between tautomers through hydrogen-bonding formation, with optimal conditions reached in the presence of two H_2_O molecules, meaning that two hydrogen bonds—established between pyrazole nitrogen and two distinct water molecules (Figure 6b)—were the most stabilizing interactions. Another goal was to understand which species carried out the donation of the first proton in the exchange process. Since pyrazoles were shown to form cationic transition states, the first species to donate the proton was found to be a water molecule. When comparing the two physical states, Oziminski suggested more synchronous exchanges in the gas phase than in solution [44].

#### 3.1.2. Experimental Studies

Several experimental techniques have been explored to study the proton transfer in pyrazoles and their derivatives, including mass spectrometry, IR spectroscopy and NMR spectroscopy [38,39,80,81,82,83,84] NMR is one of the most frequently used techniques for identification of chemical compounds and is also used to investigate chemical processes, for instance through kinetic studies. In the present context we will focus mainly on the use of NMR spectroscopy for studies related to tautomerism [39]. The advantage of NMR is that it allows the user to evaluate with accuracy the majority of factors that influence tautomeric behavior of molecules, namely the physical state, the temperature, substitution patterns and solvent effects. Hence, for studies of tautomerism in pyrazoles, measurement of chemical shifts permits accurate identification of the species present in each condition evaluated as well as the ascertainment of tautomer equilibrium constants, upon the adequate treatment of the samples and data [39]. Additionally, it has been shown that NMR data can be used for reactivity predictions in heterocyclic compounds, in light of the development of more efficacious syntheses [85].

Recent studies on tautomerism in pyrazoles based on NMR spectroscopy are mostly follow-ups to the fundamental studies disclosed in the past century, to a large extent from the valuable contributions of Elguero et al. [39,86,87,88,89,90,91,92,93,94,95,96,97]. The main conclusions of these pioneering studies were that, for pyrazole itself, in solution, a tautomeric equilibrium is observed, meaning that pyrazole exists as a mixture of two identical tautomers, with interconversion rates high enough to be undetected in the NMR time scale [89]. Hence, the same ^13^C chemical shift values were detected for the tautomeric 3 and 5 positions in DMSO-*d*_6_, in keeping with the presence of a symmetrical molecule in which the hydrogen would be shared between both nuclear nitrogen atoms [98]. This arises as a common issue in tautomerism studies of pyrazole derivatives, where a difficulty in observing individual signals for the two positions most affected by the interconversions C3 and C5 emerges, mainly in unsymmetrically substituted species. In these circumstances, the need for prototropy deceleration emanates and the main factors found to circumvent such issue were variations in the electronic nature of the ring-substituents, the physical state, the temperature and the solvent. In pyrazole itself, tautomeric interconversion rates could be successfully decreased by using dipolar aprotic solvents and low temperatures [94,95,99].

##### Substituent Effect

In early studies on the structure and tautomeric preferences of substituted pyrazoles, conducted by Cabildo et al., it was found that the ^13^C-NMR chemical shifts at the positions 3 and 5 vary according to the tautomer present in the DMSO-*d*_6_ solution, a feature that equally impacts on the chemical shifts of the carbons attached to those positions [96]. Additionally, for N-unsubstituted pyrazoles, broad signals at C3 and C5 were observed in the spectra, portraying the merging of C3 and C5 signals as a result of N1–N2 prototropic exchange and the presence of a tautomeric equilibrium. This issue was circumvented by the authors through acidification of the medium with trifluoroacetic acid (TFA), which provides a fast proton exchange, undetectable at the NMR time scale, resulting in narrow signals for the resulting species. However, although this work provided the assignment of carbon chemical shifts in pyrazoles, no clear conclusions regarding tautomeric ratios could be achieved [96].

Later, Lopez et al. studied the ^13^C-NMR spectra of a series of tautomeric pyrazoles in solution and in solid state, with the aim of uncovering the influence of variable substitution patterns, namely the chemical nature and structure of the substituents, on the tautomeric stability of pyrazoles. They reached the conclusion that general structural preferences can be associated to the chemical shifts at the 3 and 5 positions. Solution studies were carried out mainly in deuterated chloroform CDCl_3_) and/or DMSO-*d*_6_, with similar results for both solvents: monosubstituted derivatives with alkyl groups resulted frequently in a tautomeric equilibrium. Regarding the nature of the substituents, groups such as CF_3_ and heteroaromatic moieties showed preference for the 3 position whereas *tert*-butyl, isopropyl and methyl preferred carbon 5 in the selected library of compounds. As for solid state, the tautomeric prevalence exhibited high agreement with solution studies, meaning that the tautomer present in the solid state was usually the most abundant one in solution [97].

In the same year, Begtrup et al. published a compilation of the ^13^C-NMR data available, together with some new results they obtained. This work aimed essentially at the compilation of data regarding the attribution of chemical shifts in a collection of pyrazole derivatives. Coalescence of individual signals to average signals and broad band-like signals at C3 and C5 on ^13^C-NMR spectra was observed for the majority of tautomeric pyrazoles, in DMSO-*d*_6_, indicating that both tautomers are present in solution, unless low temperature analyses or solid-state measurements were performed [93]. In the solid state, NMR was identified by Faure et al. as a suitable tool for tautomerism studies, when well-resolved spectra could be obtained, showing individual structures for the majority of molecules studied [87].

Vladimir I. Minkin et al. published a work in 2000 where an exhaustive report on the studies performed to that date was presented (mostly by NMR, combined with other techniques in some cases), from which the authors proposed a trend in the tautomeric behavior of pyrazoles based on the nature of substituents at positions 3 or 5 of the pyrazole ring. For instance, in the presence of strong π-acceptor substituents, considering the position 1 as the nitrogen bearing the hydrogen atom, the preferred structure would be the one incorporating these substituents at a conjugate position from the N1 atom, i.e., the 3-tautomer. The opposite was suggested for strong σ-acceptors [50].

Claramunt et al. combined their theoretical calculations with experimental results obtained using NMR techniques, in solid and liquid environments, and crystallographic studies, to estimate the influence of different substituents on tautomerism and aggregation patterns of pyrazoles. Different aggregation patterns were predicted according to the tautomers present in each crystal—polymorphs composed of only one tautomeric structure or desmotropes with two distinct species were observed, and two types of aggregates were predicted for the analyzed compounds, dimers and tetramers (for pyrazole oligomers see Figure 3). Bulkier substituents were shown to favor dimeric crystals, while smaller ones associate into tetrameric structures [41].

##### Environmental Effect

In NMR spectroscopy studies aimed at evaluating the influence of intrinsic or extrinsic factors on tautomerism, the environment in which studies are performed must be discriminated. Solution studies on pyrazoles are highly influenced by the solvent, given their basic character, polarizability and ability to exchange protons intermolecularly, as we mentioned in a preceding segment of this manuscript. On this matter, one of the key-factors to be taken into account in solution is the putative formation of intermolecular interactions, whether they involve solely solute molecules or solute-solvent ones. On the one hand, self-assembly can have a strong impact on the concentration of the sample, influencing the NMR signals, which can be altered or lost due to solute-solvent interactions [88,100]. The solid state is also recognized for strong intermolecular interactions and many hypotheses were conceived by chemists regarding tautomerism of pyrazoles [56,87,93], thus deserving our close attention.

Solvent effects have been evaluated by Claramunt et al. in a ^15^N NMR study of several diversely substituted azoles. The most important conclusions were that nitrogen chemical shifts depend on the solvent polarity and acidity and, therefore, for this type of study, analyses should be preferentially performed in DMSO, acetone or chloroform, rather than water and alcohols [86].

Provasi et al. undertook a nematic phase ^1^H-NMR study to evaluate the tautomeric behavior of pyrazole at temperatures ranging from 299 K to 308 K, observing a fast prototropic exchange under the experiment conditions used [99].

It was previously suggested that, in solution, one way to avoid dynamic behavior leading to tautomeric mixtures in NMR spectra was to lower the analytical temperature or to perform solid-state studies, in order to block the tautomeric exchange. However, the same dynamic behavior could also be observed in the solid state. This phenomenon was firstly described by Baldy et al., following an NMR study of 3,5-dimethylpyrazole [101]. The authors have shown that broad bands at C3 and C5 on the 13C CP/MAS NMR spectrum could be well-resolved upon low-temperature analysis [101]. Later, a ^15^N NMR solid-state study of tautomerism in pyrazole-4-carboxylic acids [102], among other studies related to dynamic behavior of pyrazoles in the solid state [52], corroborated Baldy’s conclusions.

Nonetheless, it is postulated that, very often, one tautomeric form is prevalent in the solid state, and very rarely, two tautomers appear in the same crystal, as happens with catemers formed by pyrazole itself, where no dynamic behavior was observed [39]. In the aforementioned recent theoretical study, Kusakiewicz-Dawid et al. also investigated the tautomeric behavior of ester/amide 3,5-disubstituted pyrazole derivatives in the crystal phase. In fact, for all the compounds analyzed, only one tautomer was observed within each crystal lattice. The general conclusion of this study is that, according to the substituents on the ring, compound structure alters, namely the tautomeric form adopted, bond angles and intermolecular interactions leading to self-association. Additionally, in 3,5-disubstituted pyrazoles carrying a carbonyl linkage, the more electron withdrawing substituents were found to stabilize the 3-tautomer in crystals, as could also be observed in additional crystal structures collected by the Cambridge Crystallographic Data Centre [72].

## 4. Pyrazoles in Organic Synthesis

**Figure 7:**
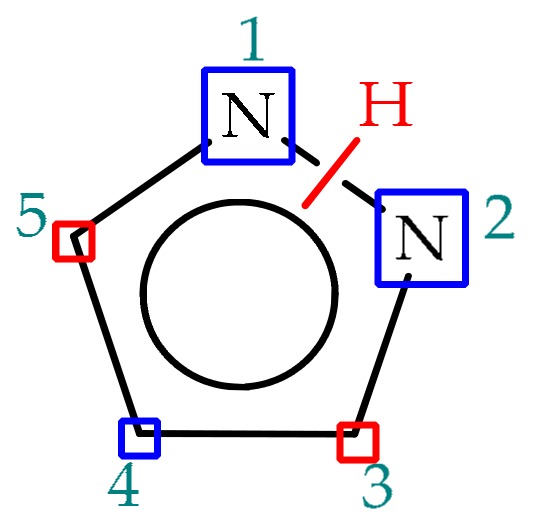
Pyrazole structure highlighting the nucleophilic and electrophilic positions in the blue and red boxes, respectively.

Pyrazoles are electron-rich heterocyclic systems which can be readily employed in organic synthesis, given their versatile chemistry. Within the pyrazole scaffold, besides the acidic and basic properties referred in the previous chapters, three positions display nucleophilic nature (N1, N2, C4) and two electrophilic ones (C3, C5), represented in Figure 7 as blue and red rectangles, respectively. This way, pyrazole functionalization may be sought at any ring position, with nucleophiles adding preferentially to C3 and C5 positions, whereas electrophile addition occurs preferentially at C4 position and/or to any of the nitrogen atoms, depending on the reaction conditions. One way to efficiently introduce substituents at annular carbons is through directed cross-coupling reactions, such as Suzuki-couplings [103], in which case C4-halogenations are usually necessary as prior steps to boronic acid or ester cross-coupling at the indicated position [104].

**Scheme 7:**
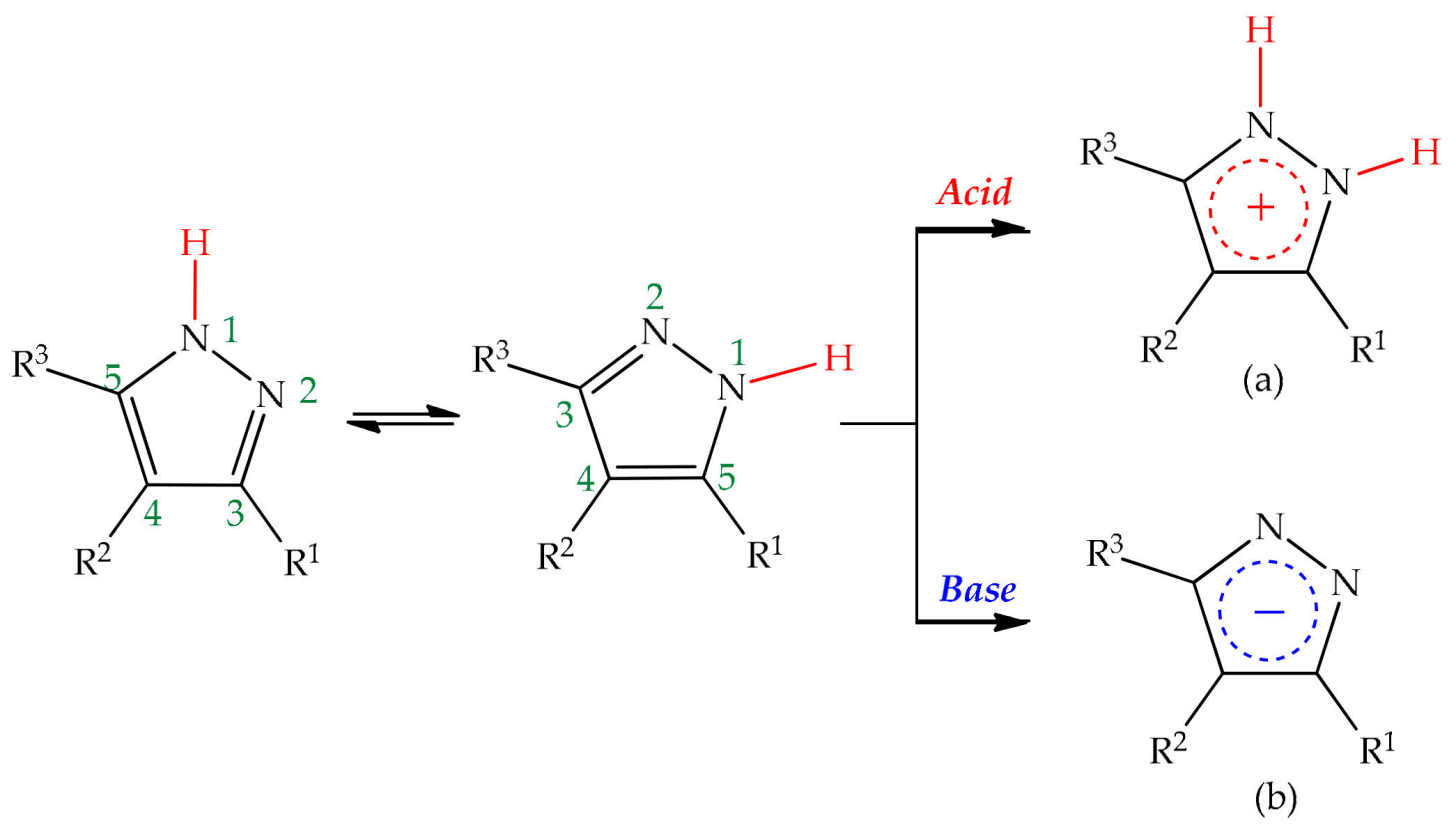
Schematic representation of the chemical behavior of pyrazoles in acid and basic media leading to pyrazole cations (**a**) and anions (**b**), respectively.

It was previously stated that, in the presence of two annular nitrogen atoms with pyrrole and pyridine-like characters, the one participating in nucleophilic substitution is the basic pyridine-like nitrogen [6]. However, it was later shown by Huang et al. that regioselective synthesis of N1 substituted pyrazoles could be achieved under specific conditions, which led them to investigate the electron density on the ring-nitrogen theoretically through density functional theory (DFT) calculations at the B3LYP/6-31G**(d) level, having concluded that the major negative charge was concentrated on the pyrrole-like nitrogen [83]. Nevertheless, organic synthesis based on pyrazole synthons should regard carefully two main aspects that strongly influence the reactivity of the molecule, the medium or environment in which reactions occur and the substitution pattern on the ring system. On the one hand, the amphoteric nature of pyrazoles makes them especially propense to protonation or deprotonation reactions in acidic and basic environments, respectively, which alters considerably the nucleophilicity of the ring by originating a positively or negatively charged pyrazole ring (Scheme 7).

The nature of substituents, namely their size and electronic contributions, may also play a role in regiochemistry, altering the overall reactivity of the system. Kost stated that electron-withdrawing groups on the pyrazole ring increase its basicity, by increasing the acidity of the proton [105]. Conversely, recent studies in 3(5)-substituted pyrazoles point to the opposite effect, with electron-donating groups at C3 increasing the basicity of the pyrazole ring [37], as shown by theoretical calculations for 3(5)-methylpyrazole [36]. Easy proton abstraction by bases generates the nucleophilic pyrazole anion (structure (b), Scheme 7), which is prone to coupling with electrophilic moieties, wherein steric constraints imposed by substituents at C3 and C5 are usually the factors determining regioselectivity [106]. Contrariwise, protonation of the pyridine-like nitrogen is also predisposed in acidic media, which in turn may direct the condensation reaction towards the participation of the exocyclic groups, as frequently happens in cyclization reactions involving 3(5)-aminopyrazoles [13,107]. Given that pyrazoles are tautomeric structures, protonation/deprotonation of the core structure could be one way to circumvent this issue, accounting for solvent and substituent properties, which often modulate reactivity and determine reaction pathways.

The versatility of the pyrazole motif offers a plethora of synthetic routes, enabling the preparation of libraries of compounds of interest to a broad community of organic and medicinal chemists. Synthetic methodologies should be carefully selected, and conditions should be optimized to favor regioselective processes. The literature continuously furnishes updates in synthetic methodologies involving pyrazoles and this topic has been covered in depth by several authors [8,19,25,26,27,108], including the meticulous and extensive review written by Fustero et al. [33].

## 5. 3(5)-Aminopyrazole

### 5.1. Tautomerism in 3(5)-Aminopyrazole

**Scheme 8:**
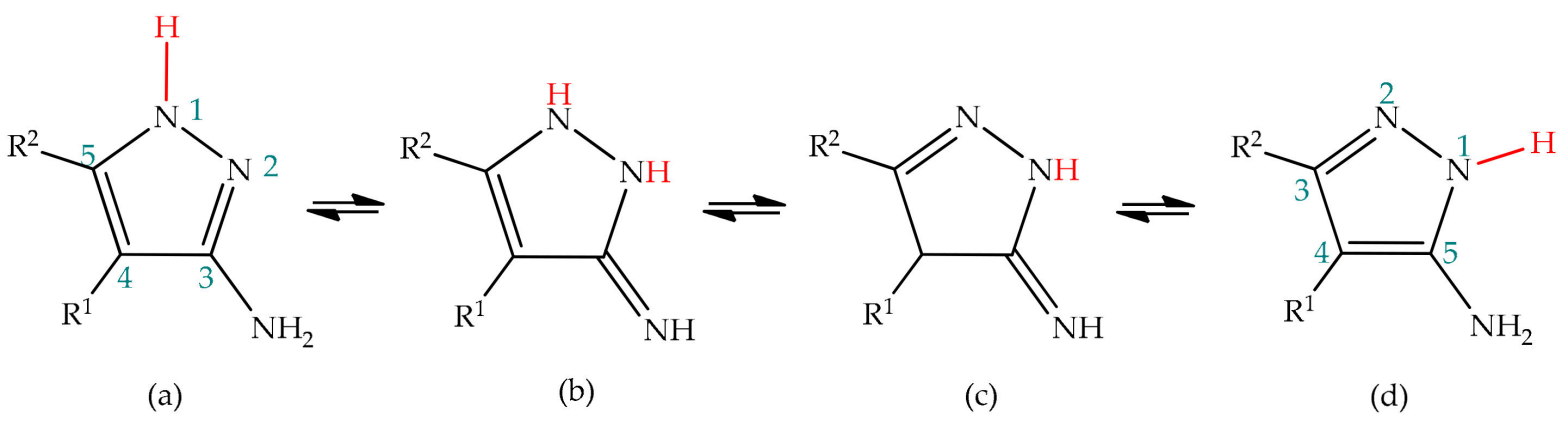
Side-chain tautomerism for 3(5)-aminopyrazole. The equilibrium between structures (**a**) and (**d**) represents the prototropic annular tautomerism for 3(5)-aminopyrazoles.

3(5)-Aminopyrazoles bear an amine substituent at the positions 3 or 5 of the pyrazole ring, as a result of prototropic annular tautomerism. In theory, the frame of 3(5)-aminopyrazole is also susceptible to side-chain tautomerism, given the presence of an amine side-chain prone to participate in proton exchange. Therefore, four tautomeric structures should be expected for 3(5)-aminopyrazoles, as depicted in Scheme 8. However, studies indicate that the imino forms are not formed, and therefore only an annular rearrangement between positions 1 and 2 is predicted for 3(5)-aminopyrazoles (structures (a) and (d) of Scheme 8), as happens for pyrazole [50,84].

Tautomerism in 3(5)-aminopyrazoles has to be carefully appraised by chemists, on account of the specificities inherent to this scaffold that impact in reactivity and synthesis. Several experimental [50,72,93,109,110,111] and theoretical studies [43,60,64,70,72,84] were carried out, the majority focusing on the structural aspects and corroborating the general conclusions applicable to the pyrazole family. Nonetheless, studies clarifying the chemistry of aminopyrazoles as reactants in organic synthesis are scarce, especially on the unsubstituted form. Further efforts are needed to enlighten the scientific community on this matter.

#### 5.1.1. Theoretical Studies

Early theoretical studies on 3(5)-aminopyrazoles relied essentially on semi-empirical methods, leaving a rather significant margin for error. One of the introductory studies of tautomerism on unsubstituted 3(5)-aminopyrazole was conducted by Catalán et al., as mentioned previously in this work. This study yielded important data regarding the basicity of the system but led to contradictory conclusions concerning gas-phase tautomeric stability. The results showed preference for the 5-amino form which, albeit being in accordance with studies published to that date, were dismissed in light of posterior research [64].

Over the years, more reliable methodologies have gradually been implemented. Abboud et al. performed an ab initio study at the HF 6-31G level of theory, also focused on the basicity of pyrazoles [112]. For 3(5)-aminopyrazole, they were able to determine a prevalence of the 3-amino tautomer in the gas phase, while a decrease in the abundance of this species was predicted in solution, owing to the higher dipole moment of the 5-amino tautomer, which becomes more susceptible to polarization by the solvent and therefore prone to more efficient solvation [112].

Higher level HF 6-311G* calculations of total energies of 3 and 5-aminopyrazoles also revealed a prevalence of 3-aminopyrazole over 5-aminopyrazole in the gas-phase, with a difference in energy between the two tautomers of 4.09 kcal/mol [60].

**Scheme 9:**
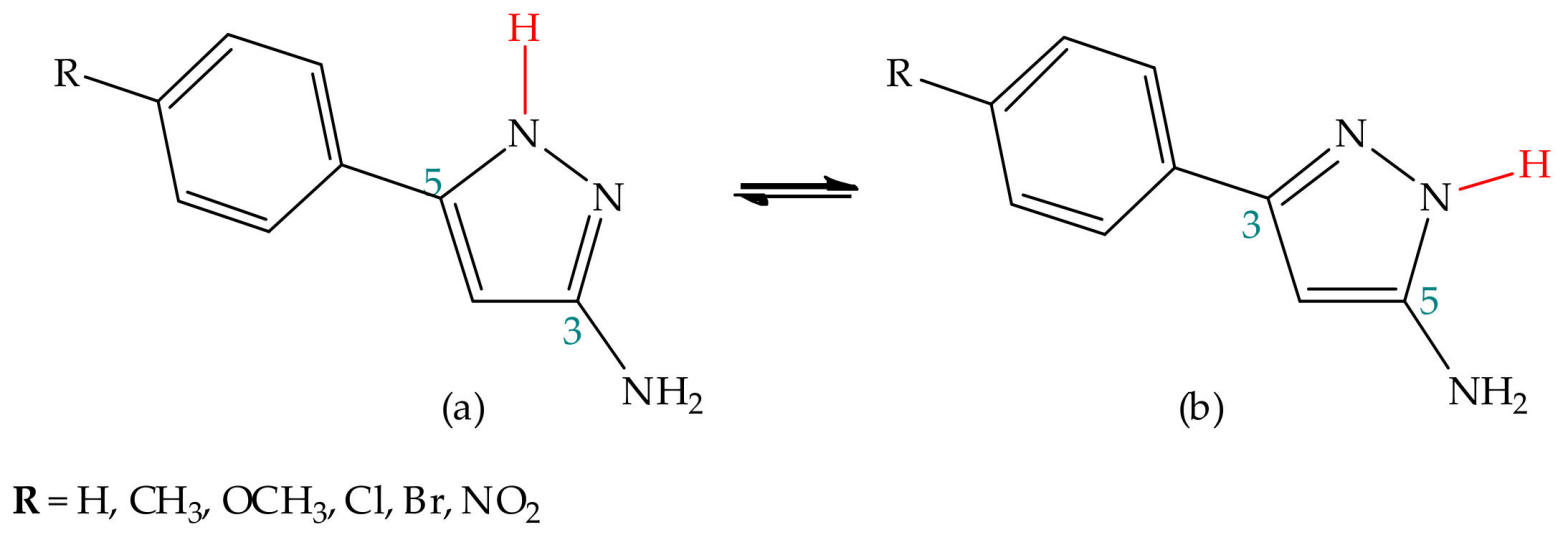
General structures of the 3(5)-amino-5(3)aryl pyrazoles studied by Puello et al. [84].

Puello et al. [84] investigated 3(5)-amino-5(3)aryl disubstituted pyrazoles differing in the nature of the substituent at the *para* position of the phenyl ring (Scheme 9). In their study, they evaluated the energetics of the tautomeric structures and, from geometry optimization at the Austin Model 1 (AM1) level, was able to conclude that, for all studied compounds, the energy for the 3-amino tautomer was lower, in overall agreement with the experimental results collected through NMR spectroscopy and X-ray crystallography studies. Nevertheless, an increase in the electron donating nature of the substituent at the phenyl ring was shown to raise the fraction of the 5-amino tautomer [84].

**Scheme 10:**
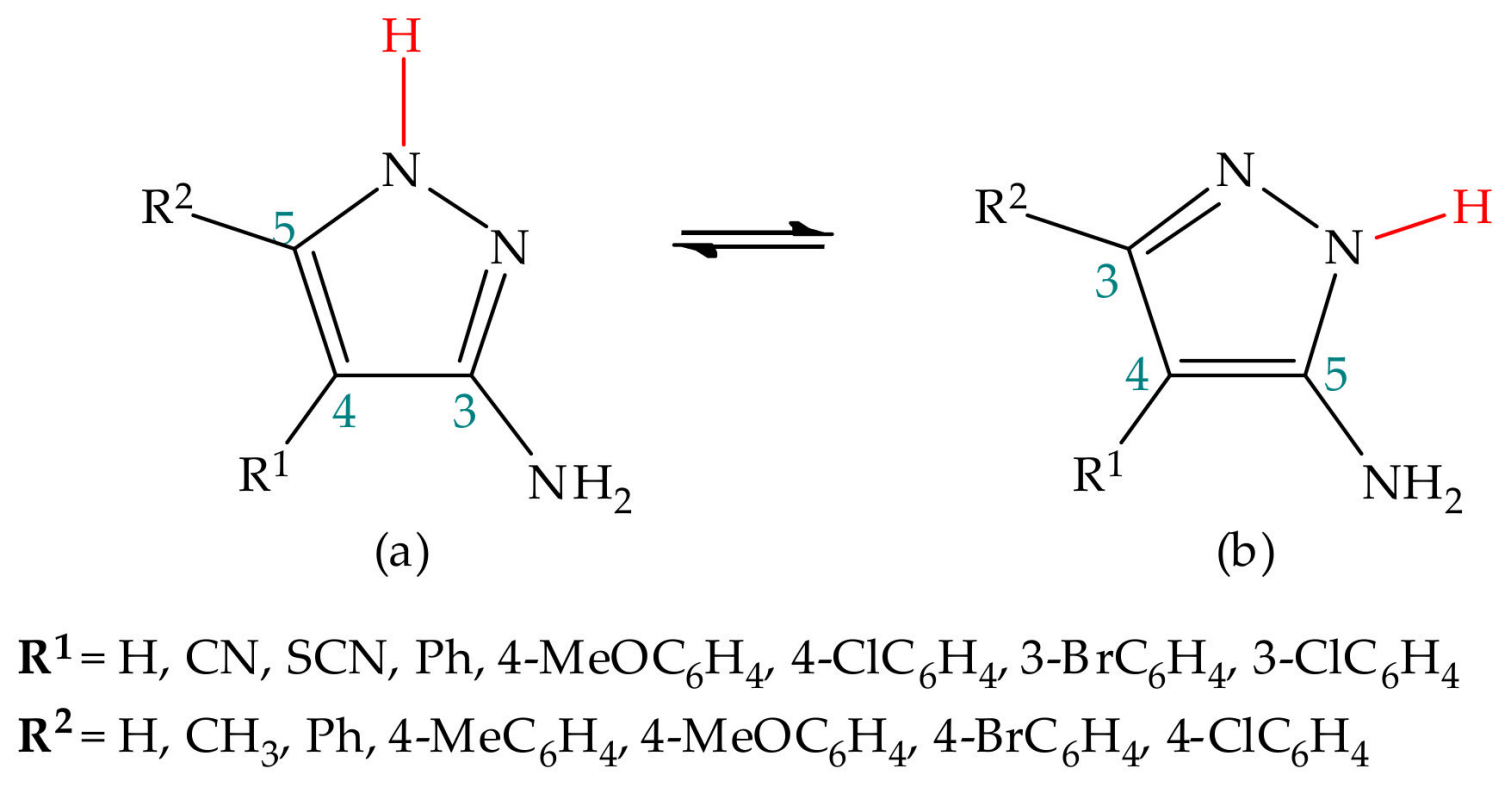
General structures of the 3(5)-aminopyrazoles studied by Emelina et al. [113].

A similar study was conducted by Emelina et al., through B3LYP/6-31G** calculations of total energies for 3-amino and 5-amino tautomers of 4-substituted 3(5)-aminopyrazoles (Scheme 10), in the gas phase and also in DMSO solution, by incorporation of the polarizable continuum model (PCM) [113]. This study clearly shows that the extrinsic factors to the nuclear aminopyrazole scaffold cannot be undermined, namely the environment and the electronic nature of the other ring-substituents. They were able to identify a higher prevalence for the 3-amino tautomer (structure (a), Scheme 10) in the gas phase, although upon introduction of electron-withdrawing substituents at C4, the ratio of the 5-amino form (structure (b), Scheme 10) was shown to increase. More significant differences were observed in DMSO solution, where the dipole moment increases for both species but more significantly for the 5-amino form. Although higher ratios of the 3-tautomer prevail, the energetic barriers between the tautomers decrease comparatively to the gas-phase. Additionally, in the presence of electron-withdrawing substituents at C4 the ratio of tautomers was shown to invert, with dominance of the 5-amino form. These results were in agreement with experimental data also collected through solid-state and solution NMR spectroscopy [113].

Jarończyk et al. performed a study at the MP2/6-311++G** level aiming at an evaluation of the structural differences between 3 and 5-aminopyrazoles and the distinct electronic contributions towards each system’s stability [66]. The main conclusion reached by the team was that a higher stability marked by the occupation of the C3 position in pyrazoles in the gaseous state derived from the positioning of the electrons at the exocyclic amino group. While in 5-aminopyrazole the lone electron pair of the amino group occupies a co-planar position with the ring, the 3-amino tautomer is favored by the additional p-electron contribution to the pyrazole nucleus of the perpendicularly positioned electrons from the NH_2_ group [66].

DFT and MP2 based calculations performed on monomeric and dimeric crystal structures of 3 and 5 substituted pyrazoles led Chermahini and Teimouri to conclude that electron donating groups at the 3-position favor the 3-tautomer, while electron withdrawing groups lead to preferential formation of the 5-tautomer. For aminopyrazole, the 3-tautomer was found to be more stable by 2.61 kcal/mol than its 5-isomer [43]. Additionally, according to the results of their MP2 calculations, the intramolecular prototropic annular tautomerism from the 3-amino to the 5-amino derivative was shown to involve an energy demand of 47.34 kcal/mol [43].

DFT 6-311++G(d,p) results attained by Marin-Luna et al. sustained the previously reached conclusions, showing a prevalence of the 3-tautomer over the 5 one, both in the gas phase and in aqueous solution, although in this case a tendency towards a tautomeric equilibrium was noticed, marked by a decrease in percentage of the 3-amino tautomer and corresponding increase of its 5-amino counterpart, ascribed to the effect of solvent polarity [70].

#### 5.1.2. Experimental Studies

For unsubstituted 3(5)-aminopyrazoles, one of the first experimental studies of tautomerism, accomplished by Dorn, was based on IR and ^1^H-NMR techniques [110]. In this study, the author found a higher abundance of the 3-amino tautomer in solution, contradicting some results obtained to that date. In another investigation, Gonzalez et al. reached similar conclusions, through NMR studies in DMSO-*d*_6_ solution complemented with some theoretical results, showing that the preference for positions 3 or 5 of the ring is mostly dependent on the electronic properties of the substituents [109].

**Figure 8:**
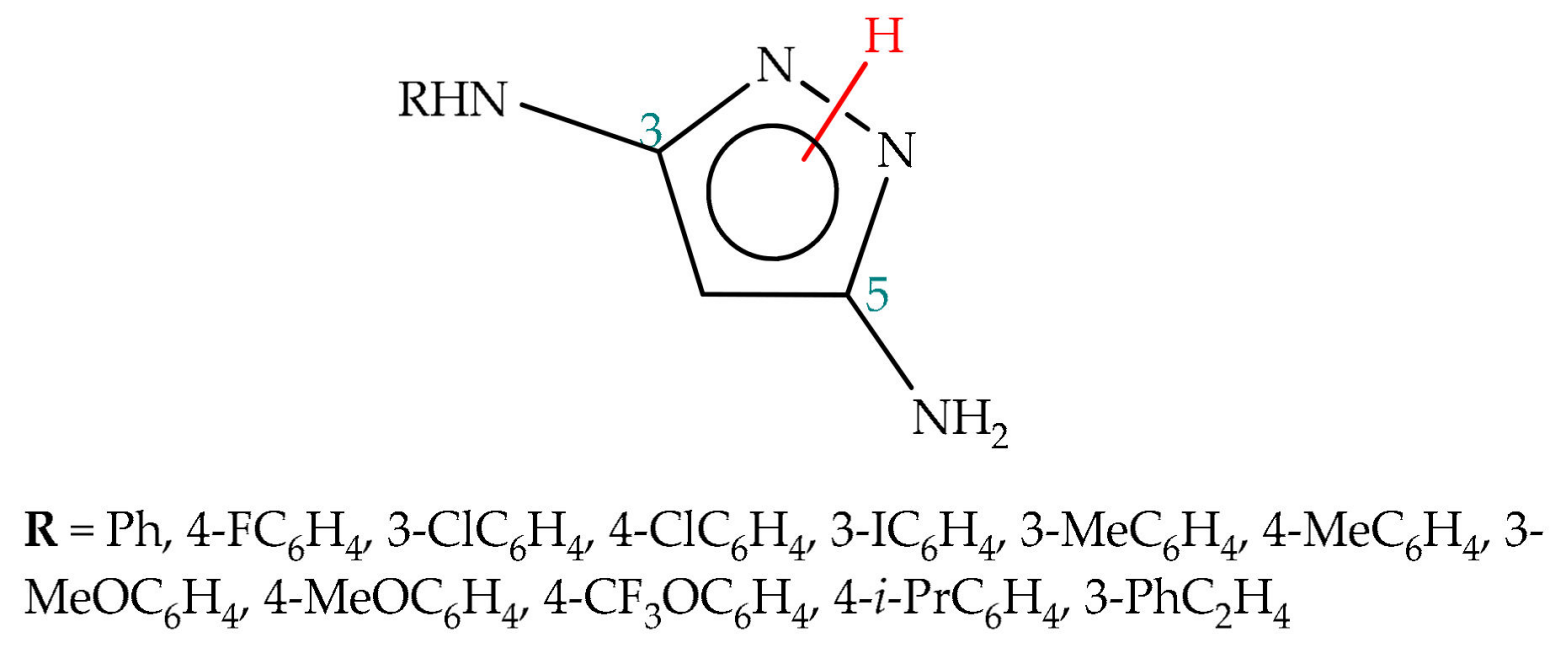
General structure of the aminopyrazole derivatives studied by Lim et al. [111].

Results of a recent investigation on 3-amino-substituted 5-aminopyrazoles (Figure 8) performed by Lim et al. [111], using solution and solid-state NMR and X-ray crystallography, were in conformity with the aforementioned properties of pyrazoles regarding proton exchange. ^1^H-NMR analysis confirmed the rapid interconversion between 3- and 5-tautomers in the presence of solvent molecules, which prevented the observation of two separate signals for the majority of molecules analyzed, but rather showed the presence of broad bands for the protons at the tautomeric positions C3/C5, as well as for the ring C-H group. Despite that, the researchers were able to observe individual structures of the tautomers for one of the compounds, 5-amino-3-phenethylaminopyrazole, which showed predominance of the form bearing the phenethylamine group, with higher electron donating properties, at the C3 position [111].

### 5.2. 3(5)-Aminopyrazole in Heterocyclic Synthesis

**Scheme 11:**
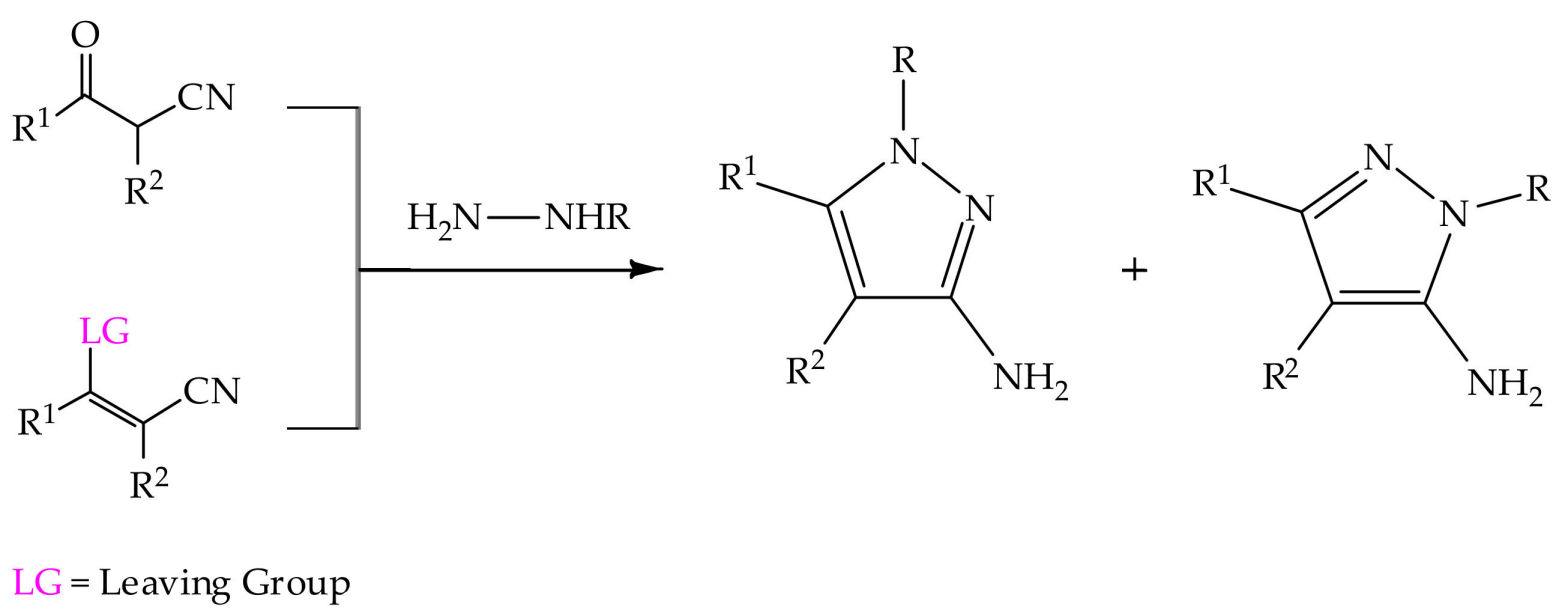
General routes to the formation of the 3(5)-aminopyrazoles from 1,3-dielectrophilic nitriles and hydrazines [20,114].

Synthesis of 3(5)-aminopyrazoles is usually carried out by incorporating the NH_2_ group in the synthons rather than functionalization of the pre-built pyrazole, which corresponds most commonly to condensation reactions of 1,3-dielectrophilic nitriles and hydrazines, since this pathway represents the most efficient and straightforward strategy (Scheme 11) [114].

The use of 3(5)-aminopyrazoles as building blocks in heterocyclic synthesis is challenging, mostly because these are polyfunctional compounds exhibiting a high chemical variability within the structure. 3(5)-aminopyrazoles are composed of one electrophilic C3/C5 position, depending on the considered tautomer, and four main nucleophilic sites: the two ring nitrogen atoms, the 4-carbon and the exocyclic amine. These characteristics must be duly considered in the design of synthetic methodologies, since for example the nucleophilic positions display distinct reactivities, i.e., while the C4 carbon participates in electrophilic aromatic substitutions, the nitrogen atoms mediate typical S_N_2 reactions. These properties have been widely explored in the synthesis of fused heterocycles [107], which is of our particular interest, in view of the construction of pyrazolo[1,5-a]pyrimidines.

Recent reviews cover synthetic methodologies using 3(5)-aminopyrazoles as buildings blocks to the present state of the art [13,16,107]. However, the majority of these works fail to explain the discrepancies noted in somewhat similar conditions and reactivity aspects linked to the tautomeric precursor are elusive. For this reason, our focus in this review is on the chemical potential of 3(5)-aminopyrazoles in heterocyclic synthesis. As stated by El-Sattar et al., the chemical potential of a compound reflects the relationship between its structure and reactivity, thus a comparatively higher chemical potential of a compound means a higher reactivity [115]. In compounds that exhibit tautomerism, the reactivity may be controlled by a particular tautomeric form and, according to the Gustafsson paradox, the less abundant tautomer tends to be the most reactive one [116]. In these circumstances, a paramount need to elucidate the precise chemical potential of the entities present in a specific reactant mixture emerges. HOMO and LUMO states and energies of the tautomeric structures represent the parameters that mostly contribute to their chemical potential; as such, variations alter the acidic/basic and electrophilic/nucleophilic properties of the compounds [85,115]. It was already shown that in heterocycles, including 3(5)-aminopyrazoles, the different characteristics of molecular orbitals promote distinct reactivities of each of the tautomeric species [66,85]. For 3(5)-aminopyrazole, from the information gathered in the present work, and given the nature of the NH_2_ substituent, one may infer that the 3-tautomer should be the most stable one. Conversely, the majority of synthetic strategies to produce more complex heterocyclic systems rely on the 5-tautomer to be pursued.

#### 5.2.1. Intramolecular Cyclization of 3(5)-Aminopyrazole

**Scheme 12:**
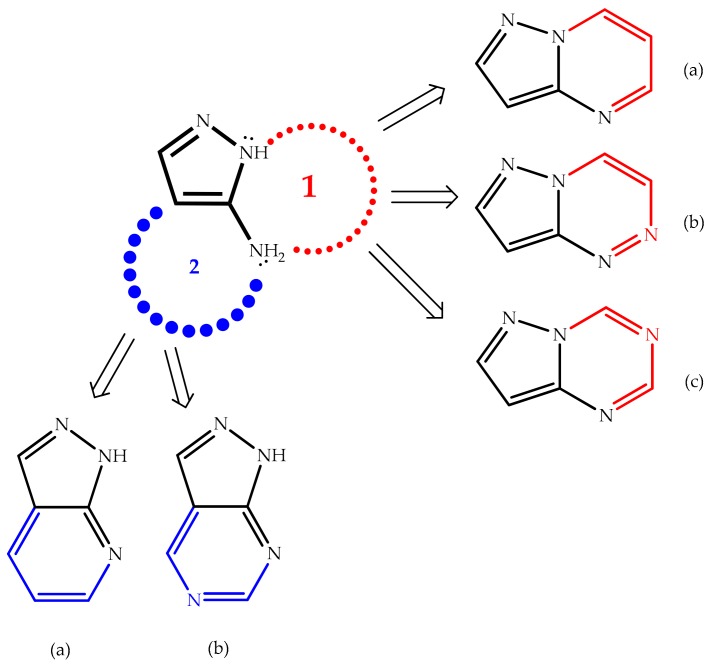
Schematic representation of the two possible pathways to intramolecular cyclization from 3(5)-aminopyrazoles. Cyclization through pathway 1 starts from the N(H)1/NH_2_ dinucleophilic centers to promote formation of (**a**) pyrazolo[1,5-a]pyrimidines, (**b**) pyrazolo[5,1-c]-1,2,4-triazines, (**c**) pyrazolo[1,5-a]-1,3,5-triazines. Pathway 2 employs the C4/NH_2_ dinucleophilic centers to give structures (**a**) pyrazolo[3,4-b]pyridines and (**b**) pyrazolo[3,4-d]pyrimidines.

When considering the 3(5)-aminopyrazole scaffold as a building block for the construction of fused heterocycles, the structural and reactivity findings have to be present. Depending on the chemotype targeted, several pathways may be thought by the organic chemist [13,15], but for the purpose of this work we will focus mainly on the preparation of pyrazole-fused six-membered heterocycles from coupling of 3(5)-aminopyrazoles with bifunctional reagents. In such terms, intramolecular cyclization relies on the participation of two of the nucleophilic positions from the aminopyrazole framework, one of them being the exocyclic amino moiety, which was shown to occupy the leading position in reactivity [18,107,113]. Aside from the exocyclic amino group, another nucleophilic element from the ring system is needed to promote ring closure. In this step, either one ring nitrogen can participate, leading to structures such as pyrazolo[1,5-a]pyrimidines, pyrazolo[5,1-c]-1,2,4-triazines, pyrazolo[1,5-a]-1,3,5-triazines, etc. or the nucleophilic carbon located at position 4, leading to fused systems such as pyrazolo[3,4-b]pyridines and pyrazolo[3,4-b]pyrimidines, among others (Scheme 12) [13,15,107,117,118,119,120,121].

**Scheme 13:**
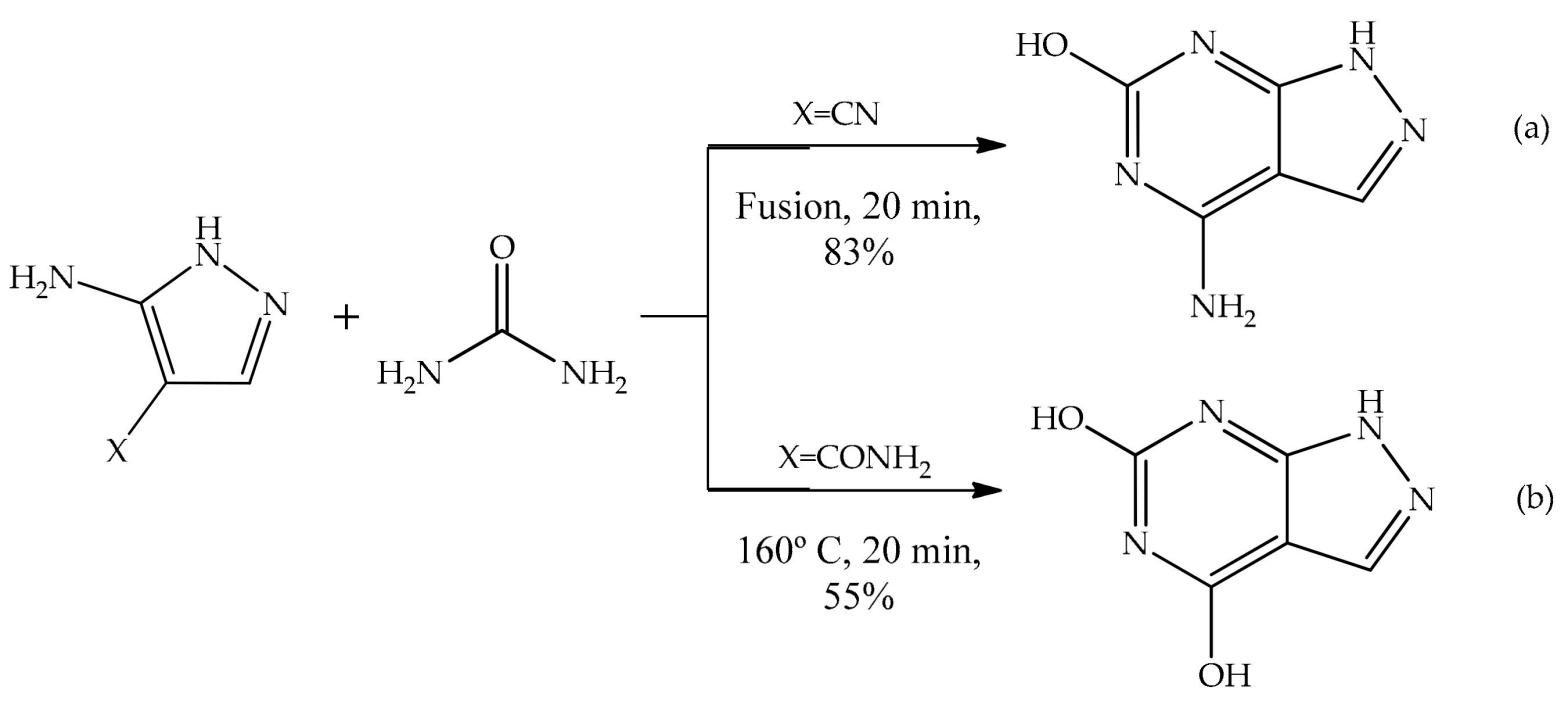
Synthesis of pyrazolo[3,4-d]pyrimidines (**a**,**b**) from 4-cyano and 4-amido substituted 5-aminopyrazoles, respectively, and urea [118]. Synthesis of pyrazolo[1,5-a]pyrimidines.

The duality in reactivity offered by the 3(5)-aminopyrazole frame towards 1,3-dielectrophilic agents demands a close attention to the synthetic methodologies in pursuit of chemoselectivity, as for example C4/NH2 cyclization products (pathway 2 of Scheme 12) are favored upon introduction of substituents at N1 [122] and the opposite effect occurs upon substitution at position 4 carbon, favoring the formation of N(H)1/NH2 products (pathway 1 of Scheme 12) [117]. However, C4 substituents must be considered attentively in the synthesis of bicyclic fused pyrazoloazines from 3(5)-aminopyrazoles since activation promoted by electrophilic or nucleophilic substituents may assist intramolecular cyclization through pathway 2 (Scheme 12). One good example of such would be the synthesis of pyrazolo[3,4-d]pyrimidines from 5-aminopyrazoles carrying nitrile or amide substituents at C4 and urea (Scheme 13) [118]. The reaction unfolds in this manner given the presence of electrophilic groups at C4 prone to suffer nucleophilic substitution by the second reagent whenever the latter possesses a sufficiently nucleophilic moiety to promote the condensation, besides the electrophilic one attacked by the exocyclic amine from 5-aminopyrazole.

Pyrazolo[1,5-a]pyrimidines belong to a class of compounds of noteworthy interest for organic and medicinal chemists [17,31,123,124,125,126,127]. As the name suggests, this chemotype results from fusion of two heterocyclic systems: the pyrazole ring and the pyrimidine ring. From a synthetic point of view, the pyrazolo[1,5-a]pyrimidine scaffold can be obtained by employing different strategies [15,16,17], including the condensation of the 3(5)-aminopyrazole precursor with 1,3-dielectrophilic agents.

**Scheme 14:**
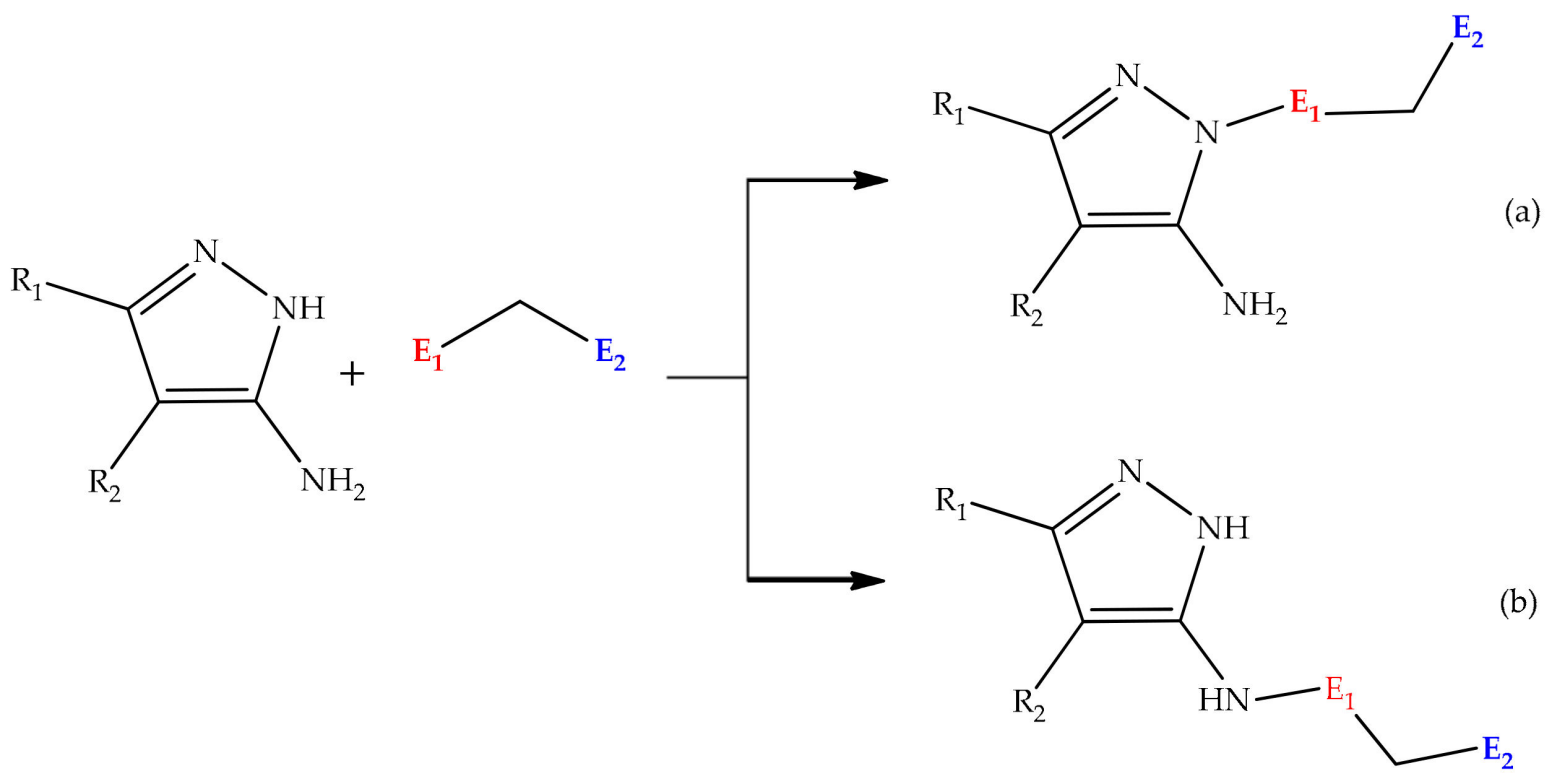
Schematic representation of S_N_2 pathways leading to the N-*endo* (**a**) and N-*exo* (**b**) products, from coupling of 5-aminopyrazole with the 1,3-dielectrophilic unsymmetrical system, where E_1_ represents the most reactive electrophile and E_2_ the least reactive one.

N-unsubstituted 3(5)-aminopyrazoles are tautomeric species possessing three N-nucleophilic sites, -NH_2_, N(H)1 and N2, which can readily participate in S_N_2 reactions leading to N-endocyclic and N-exocyclic products, as depicted by the structures (a) and (b) of Scheme 14 [128]. In terms of electronic distribution, the major electron density is located at the exocyclic amino group, in both 3 and 5 tautomers, followed by the pyrrole-like nitrogen, the pyridine-like nitrogen and the 4-carbon [113]. Although literature reports state that 3(5)-aminopyrazoles are generally more stable as the 3-tautomer, intramolecular cyclization to yield pyrazolo[1,5-a]pyrimidines depends on the formation of the most reactive form, the 5-tautomer [128]. The fused heterocycle results from the reaction of the two most nucleophilic nitrogen atoms of 3(5)-aminopyrazoles with a variety of dielectrophilic systems. In order to obtain intramolecular cyclization from the two reactant species, the nucleophilic nitrogen atoms of the pyrazole derivative have to be adjacent, favoring the 5-tautomer in this synthetic scheme.

Note that although a common pattern should be expected in the synthesis of pyrazolo[1,5-a]pyrimidines from 5-aminopyrazoles, usually different products may result from rather similar conditions [129]. Cyclocondensation of 5-aminopyrazoles with dielectrophiles are two-step reactions, where one of the nitrogen atoms should react firstly, followed by the second one, succeeding the elongation of the side-chain. This feature is important for regiochemical purposes, as for example isomeric mixtures are occasionally yielded from unsymmetrical dielectrophiles. Thus, two possible pathways may be followed, the one in which the first step is promoted by the *endo*-N and the one where the *exo*-N leads to the formation of the intermediate adduct (Scheme 14).

Regarding the nucleophilicity and reactivity of the nitrogen centers involved in this cyclization, divergent conclusions are presented in the literature. While in some synthetic schemes authors defend that the endocyclic nitrogen is the most nucleophilic center, the opposite is observed in others [130,131], indicating that regioselectivity is affected by the substituents and the reactional medium.

#### 5.2.2. Substituent Effect

When discussing the substituent effect in 5-aminopyrazoles as building-blocks, two aspects should be taken into consideration. On the one hand, in the tautomeric species, substituents play a leading role in tautomeric stabilizations. On the other hand, as a divalent scaffold, substituents may direct the reactions pathway, leading to regiospecific products.

We have mentioned chemistry studies of pyrazoles indicating that preferential occupation of the 3 or 5 positions of the ring is dependent upon the electronic properties of the substituents, with electron donating groups generally stabilizing the 3-tautomer whereas electron withdrawing groups increase the ratio of 5-tautomer. In aminopyrazoles, the NH_2_ substituent carries electron donating properties, predisposing the formation of 3-aminopyrazoles to the detriment of its tautomeric analogue, independently of the physical state. However, substituents in 3,5-disubstituted pyrazoles were shown to compete for a specific position, owing to electronic, spatial and/or geometric properties, as well as substitutions at the C4 ring position, which means that the structure of 3(5)-aminopyrazoles carrying additional ring-substituents should be carefully considered in view of the construction of pyrazolo[1,5-a]pyrimidines from the 5-amino tautomer. Moreover, steric and electronic factors from the ring substituents modulate the reactivity/nucleophilicity properties of the N(H)1/NH_2_ dinucleophilic centers, as for example electron donation towards the ring augments the reactivity of the annular nitrogen, favoring the *endo*-N pathway, whereas the exocyclic attack is favored sterically.

#### 5.2.3. Environmental Effect

3(5)-Aminopyrazoles are structural acids and bases, given their amphoteric nature provided by the nitrogen atoms composing the pyrazole nucleus. In the presence of acidic or basic medium, protonation or deprotonation of the ring nitrogen yields a charged ring-system as shown in Scheme 7 of the previous chapter, which channelizes the synthesis towards a regioselective pathway. A basic environment will generate a heterocyclic anion, enhancing the nucleophilicity and reactivity of the ring nucleophiles, whereas an acidic medium will stimulate the exocyclic attack by affording a cationic nucleus. For this reason, acid/base catalysis is usually the preferred methodology to promote selectivity in the synthesis of pyrazolo[1,5-a]pyrimidines from aminopyrazole derivatives.

#### 5.2.4. Acid Catalysis

In 3(5)-aminopyrazoles, two positions are potentially susceptible to protonation in an acid medium, the pyridine-like endocyclic nitrogen and the exocyclic amine group. Studies have shown that these compounds behave as typical heterocyclic bases, which means that protonation occurs more readily at the ring nitrogen instead of the exocyclic amine, generating the pyrazolium ion [64,98,112]. A double protonation would still be plausible, given the presence of a mildly basic amine substituent, but this possibility was abandoned when Bruix et al. [98] were able to show that a positive charge within the pyrazole nucleus deactivates the *exo*-NH_2_ group, inhibiting further protonation, independently of the strength of the acid. In these conditions, unless a stronger nucleophile is present at any of the other positions of the pyrazole ring, nucleophilic addition via *exo*-NH_2_ takes place as an initial step of the reaction, proceeding with cyclocondensation via *endo*-N nucleophilic attack to the second electrophilic moiety.

#### 5.2.5. Base Catalysis

In order to obtain cyclization by means of the endocyclic nitrogen atoms, basic medium should be preferably used, so that electron density in the ring system can contribute to the higher nucleophilicity of the system. In these conditions the conjugate base is formed, the pyrazolide anion, and the attack to the electrophile occurs at that position as an initial step of the cyclization reaction, followed by ring-closure mediated by the exocyclic amino group. Nevertheless, a general rule cannot be established for 3(5)-aminopyrazoles, since the reactivity is highly modulated by the electronic and steric features of the substituents. A notable example to the impact of such effects is provided by 3(5)-amino-5(3)-hydroxy pyrazoles, in which the ring is stabilized through resonance by the -OH group, resulting in favorable formation of pyrazolo[1,5-a]pyrimidines in acid media and pyrazolo[3,4-b]pyrimidines in basic media [15]. Thus, each case demands specific attention and structural characterizations should be conducted to confirm the intended target structures in a synthetic scheme.

## 6. Conclusions

Pyrazoles are multifarious frameworks with chemical diversity, which makes them peculiarly interesting for the organic chemist, whether in the structure elucidation aspect, or the chemistry and reactivity profile as building blocks for more complex heterocyclic systems. In this work we provide a detailed insight on the aforementioned features based on the literature developments reached thus far, but a strong need for more structural and chemical elucidations remains. Future efforts are required for the advancement of more precise chemistry based on pyrazoles.

## Figures and Tables

**Table 1:** Tautomer ratios calculated [41] for molecules 1–6 at 298.15 K.

Pyrazole	R^1^	R^2^	% Tautomer (a)	% Tautomer (b)
1	Phenyl	Methyl	66%	34%
2	Phenyl	Ethyl	63%	37%
3	Phenyl	Trifluoromethyl	27%	73%
4	Phenyl	Isopropyl	58%	42%
5	Phenyl	Benzyl	72%	28%
6	Benzylphenyl	Trifluoromethyl	0.6%	99.4%
